# Ecology and resistance to UV light and antibiotics of microbial communities on UV cabins in the dermatology service of a Spanish hospital

**DOI:** 10.1038/s41598-023-40996-8

**Published:** 2023-09-04

**Authors:** Esther Molina-Menor, Nicolás Carlotto, Àngela Vidal-Verdú, Amparo Pérez-Ferriols, Gemma Pérez-Pastor, Manuel Porcar

**Affiliations:** 1 Institute for Integrative Systems Biology (I2SysBio, University of Valencia-CSIC), Valencia, Spain; 2 Servicio de Dermatología, Consorcio Hospital General de Valencia, Valencia, Spain; 3 Darwin Bioprospecting Excellence SL (Parc Científic Universitat de València, C/ Catedràtic Agustín Escardino Benlloch 9, Paterna, Spain

**Keywords:** Microbial communities, Metagenomics, Microbial ecology, Microbiome, Policy and public health in microbiology

## Abstract

Microorganisms colonize all possible ecological habitats, including those subjected to harsh stressors such as UV radiation. Hospitals, in particular the UV cabins used in phototherapy units, constitute an environment in which microbes are intermittently subjected to UV irradiation. This selective pressure, in addition to the frequent use of antibiotics by patients, may represent a threat in the context of the increasing problem of antimicrobial resistance. In this work, a collection of microorganisms has been established in order to study the microbiota associated to the inner and outer surfaces of UV cabins and to assess their resistance to UV light and the antibiotics frequently used in the Dermatology Service of a Spanish hospital. Our results show that UV cabins harbor a relatively diverse biocenosis dominated by typically UV-resistant microorganisms commonly found in sun-irradiated environments, such as *Kocuria*, *Micrococcus* or *Deinococcus* spp., but also clinically relevant taxa, such as *Staphylococcus* or *Pseudomonas* spp. The UV-radiation assays revealed that, although some isolates displayed some resistance, UV is not a major factor shaping the biocenosis living on the cabins, since a similar pool of resistant microorganisms was identified on the external surface of the cabins. Interestingly, some *Staphylococcus* spp. displayed resistance to one or more antibiotics, although the hospital reported no cases of antibiotic-resistance infections of the patients using the cabins. Finally, no association between UV and antibiotic resistances was found.

## Introduction

Microorganisms (not only bacteria and archaea, but also eukaryotes) can resist both ultraviolet radiation and high doses of ionizing radiation^1^. UV-resistant organisms are widely distributed in many ecosystems, but they are particularly frequent on sun-irradiated environments such as building surfaces, deserts or solar panels^2–4^.

Radiation-resistant bacteria belong to different taxonomic groups. Although some clades are known by their natural resistance to radiation and high temperatures, such as the *Deinococcocota* phylum (former *Deinococcus-Thermus*), radiation-resistant bacteria are also represented in *Pseudomonadota*, *Bacillota*, *Actinomycetota* or *Bacteroidota*, previously *Proteobacteria*, *Firmicutes*, *Actinobacteria* and *Bacteroidetes*, respectively^5^. In addition to their general non-pathogenic condition, they can even contribute to other organisms, such as plants, fighting pathogenic microorganisms^6^.

Pathogenic bacteria, in particular those causing health problems to humans and other animals, show different sensitivities to UV radiation^7^. In fact, irradiation is used as an antimicrobial strategy in different laboratory and hospital devices such as UV sterilization lamps and microbiology cabins^8^. Besides, UV light and other light-based strategies have been also proposed as potential strategies to treat microbial infections in patients^9, 10^. However, UV sterilization has some limits as its efficiency depends on factors such as microbial species and state of cultures, or the nature of the surfaces, among others^7, 11^.

UV light covers the spectrum wavelength between 100 and 400 nm. It can be further subdivided into three regions: UVA (315–400 nm), UVB (280–315 nm) and UVC (100–280 nm)^12^. Each UV light range has a different effect on living organisms, being UVC the most energetic, and thus dangerous, radiation. However, solar radiation that reaches the Earth’s surface is UVB and UVA, as UVC is absorbed by the atmosphere^13^. Although UVB is also mainly filtered, the small fraction that gets to the surface causes different deleterious effects on the organisms, such as skin tanning and sunburns in animals. UVB light is directly absorbed by DNA molecules causing mutations, which is associated with the development of several types of skin cancer. Moreover, it is major responsible of killing airborne bacteria subjected to sunlight^14^. In contrast, UVA light is the most penetrating one and represents 95% of the UV light that reaches our planet’s surface. Its penetrating power has an impact on photoaging, but it can also contribute to DNA damage by interacting with already existing photoproducts^13, 15, 16^.

The damaging effects of UV light is both direct (changes in biomolecules) and indirect, via the increase in reactive oxygen species (ROS). It includes changes in DNA, such as the formation of pyrimidine dimers, but also structural changes in proteins, lipids, and physiological stress that leads to loss of cell viability^16–18^. Interestingly, and beyond the antiseptic effect of UV treatment described above, both UVB and UVA, at controlled doses, can be used for therapeutic purposes to treat cutaneous affections.

Phototherapy is the controlled use of light of different wavelengths to treat health problems, mostly skin disorders^19^. It is commonly used in new-borns developing jaundice, to treat the accumulation of bilirubin^20^, but also in the treatment of psoriasis, chronic eczema, mycosis fungoides or vitiligo, among other diseases^21, 22^.

Antimicrobial resistance, AMR, has risen as one of the main threats for global health. Specifically, the World Health Organization (WHO) estimates that by 2050, around 10 million people will die from infections with no available treatments^23^. Although the selection of resistances is a natural phenomenon resulting from the imposed selective pressure of using antibiotics, their abuse and misuse, among other factors, has accelerated their spread^24, 25^. Specifically, hospitals and intensive care units (ICUs) represent environments in which the risk of acquiring nosocomial multidrug resistant infections increases^26^. The systematic use of antibiotics in hospital environments, in addition to the abundance of more susceptible patients, favours the spread of AMR among microorganisms^27^.

The studies regarding AMR and radiation mainly focus on the effect of IR on the degradation of both antibiotic products and the inactivation of gene synthesis^28^, or the treatment of infections with specific IR^29^. Although there is some evidence that the use of radiation may favour the selection of antibiotic resistant bacteria, there are no previous reports on their study in phototherapy services^30^.

In the present work, we aimed at studying the microbial diversity in a previously unreported niche: therapeutic UV light hospital cabins, and to explore whether there is a link between the origin of the samples (taken either inside or outside the cabins) and UV resistance. Additionally, we have explored the co-occurrence of UV resistance and antibiotic resistance. Such co-occurrence may be of special interest in order to inform UV-based antimicrobial and disinfection policies in hospital facilities^26, 31^.

## Results

### Ecology and microbial diversity

#### Culturable microbial collection and identification

**Figure 1:**
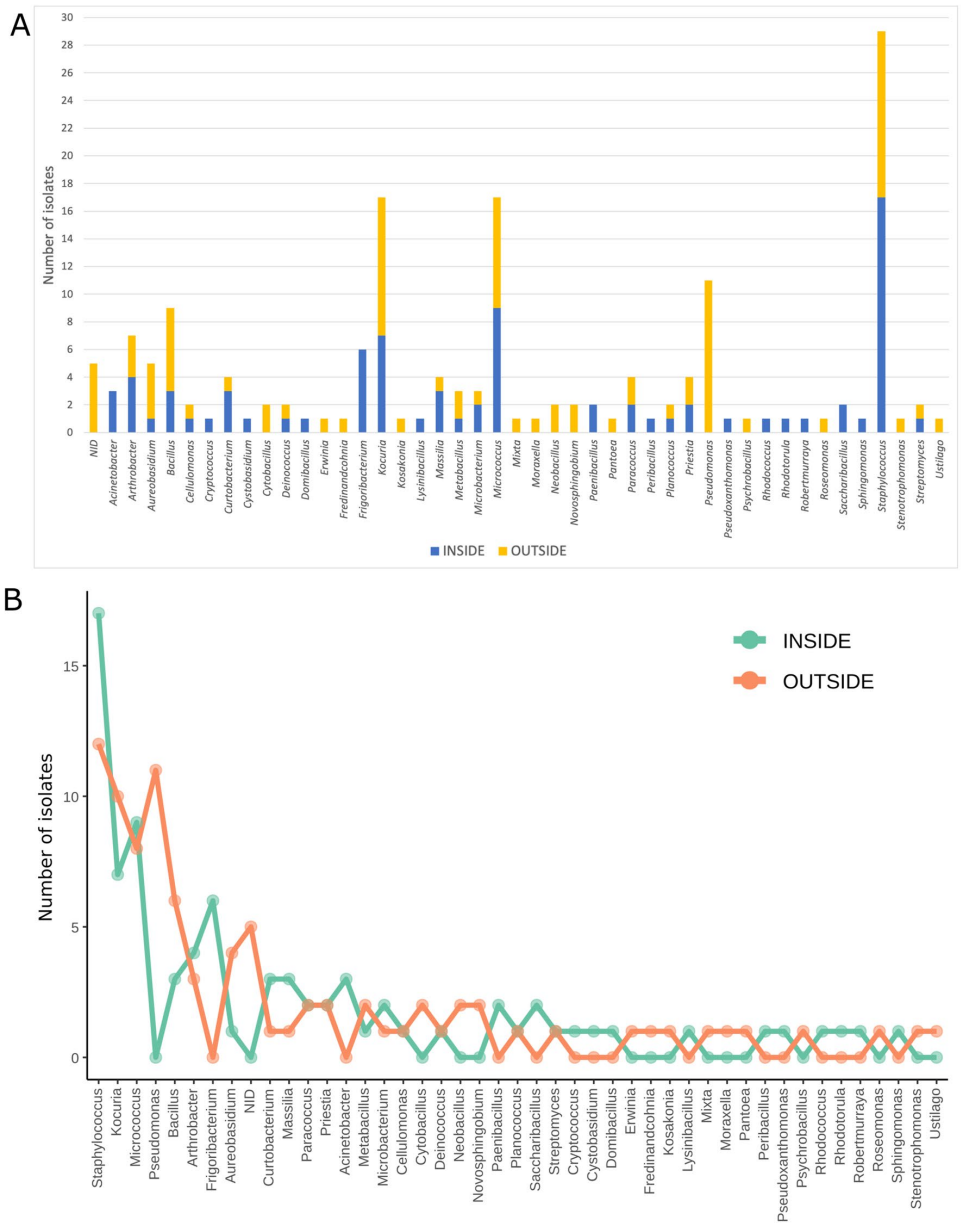
Culturable microbial diversity. (**A**) Histogram showing the number of strains isolated from inside (blue) and outside (yellow) the cabins. The genera are listed in alphabetical order. Non-identified isolates (NID) are also included. (**B**) Lineplot representing the number of strains isolated from inside (green) and outside (orange) from the most abundant to the less abundant.

The establishment of a microbial collection resulted in the isolation of 169 strains. A total of 164 isolates were identified, of which 155 corresponded to bacterial species. The identification through 16S rRNA gene or ITS sequence sequencing revealed that the isolates belonged to 44 different genera, among which the genera *Staphylococcus* (29 isolates), *Kocuria* (17 isolates), *Micrococcus* (17 isolates) and *Pseudomonas* (11 isolates) were the most abundant ones. In contrast, genera *Erwinia*, *Fredinandcohnia*, *Lysinibacillus*, *Mixta*, *Moraxella*, *Peribacillus*, *Pseudoxanthomonas*, *Rhodococcus*, *Robertmurraya*, *Roseomonas*, *Pantoea*, *Psychrobacillus*, *Domibacillus*, *Kosakonia*, *Ustilago*, *Cryptococcus*, *Cystobasidium* and *Rhodotorula* were just represented by one isolate each (Fig. 1A,B).

**Figure 2:**
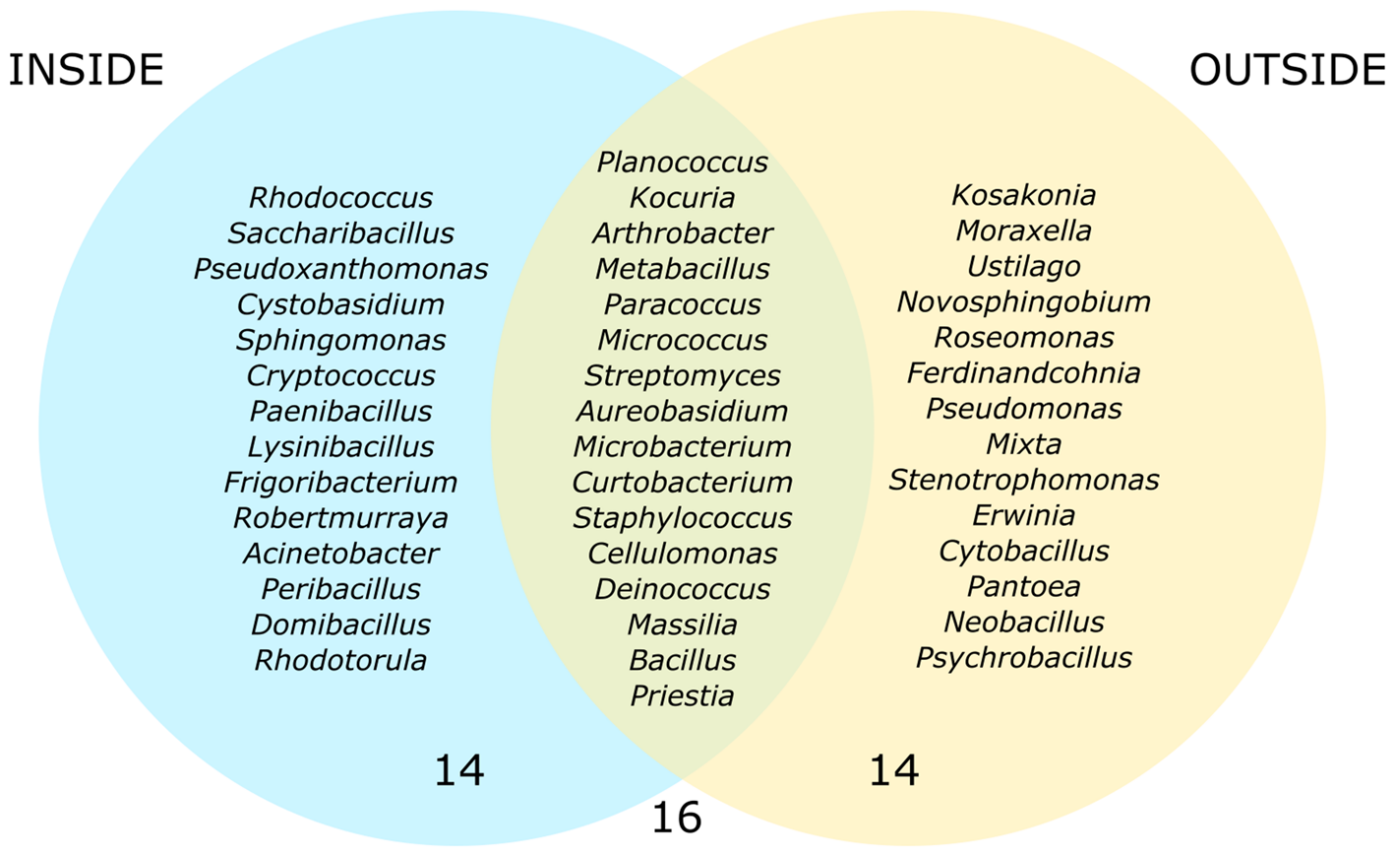
Venn diagram showing the exclusive and shared cultured genera isolated from inside and outside the cabins.

Taking into consideration the isolation source, 81 isolates originating from the inner surface of the cabins, being *Staphylococcus* (17 isolates) the most abundant genera. In contrast, 88 microbial isolates came from the outer surface of the cabins, among which *Staphylococcus* (12 isolates), *Pseudomonas* (11 isolates) and *Kocuria* (10 isolates) were the most abundant ones. Moreover, 14 genera were exclusively isolated from each location (inside and outside), whereas 16 were common for both sampling sites (Fig. 2). However, it has to be stressed that most of the “exclusive” taxa were represented by just one isolate, with the exception of *Pseudomonas* spp. When comparing the cabins, the four most abundant genera (*Staphylococcus*, *Kocuria*, *Micrococcus* and *Pseudomonas*), as well as *Bacillus*, were isolated from all of them and cabin two was the one with the highest number of exclusive taxa (11) regardless of the isolation source (Fig. S1C). However, the exclusivity inside and outside was similar (Fig. S1A,B).

#### Next generation sequencing: high-throughput 16S rRNA gene sequencing

**Figure 3:**
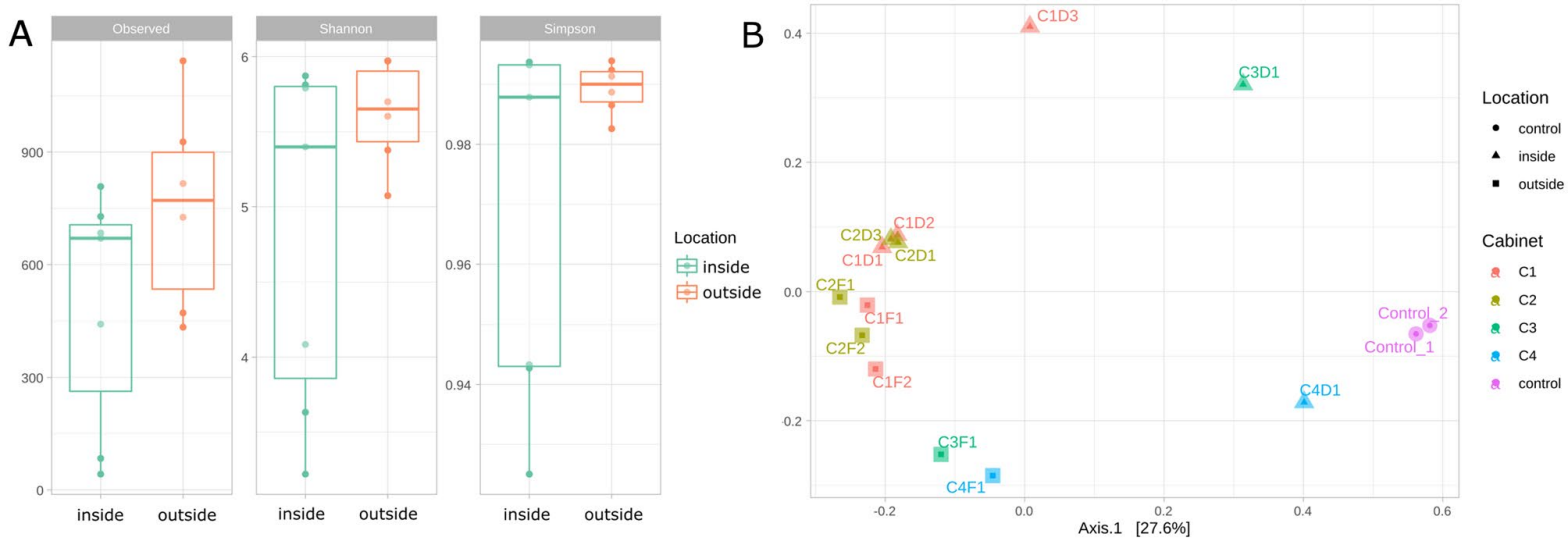
Microbial diversity in the cabins. (**A**) α-diversity at the ASV level (clones) observed through Wilcoxon test and measured by Shannon and Simpson indexes. (**B**) PCoA showing the β-diversity of the samples from four different cabins both from inner and outer surfaces. Control samples corresponding to the DNA extraction kits used are included.

Three different α-diversity indexes were calculated: richness, Shannon index and Simpson index. Richness refers to the total number of amplicon sequence variants (ASVs, or clones), Shannon measures the number of different taxa and their abundances, whereas Simpson quantifies how the sequences are distributed among ASV. That is, the number of bacteria per ASV. Although the α-diversity (ASV level) was higher in the samples from the outside than from the inside of the cabins, the Wilcoxon test did not find significant differences given the low number of replicates from each cabin and location. Similarly, the Shannon and Simpson indexes revealed higher diversity values for the outside of cabins two, three and four, but again, these results were not significantly different (Figs. 3A, S3A,B).

The representation of the β-diversity in a principal component analysis (PCoA) showed that the outer surfaces of the cabins displayed higher similarities than the inner surfaces among cabins. Moreover, samples from both the inside and the outside of cabins one and two were similar in terms of microbial composition but plotted separately in the PCoA (Fig. 3B). The PERMANOVA test confirmed that the microbiomes were significantly different both between cabins and sample locations.

**Figure 4:**
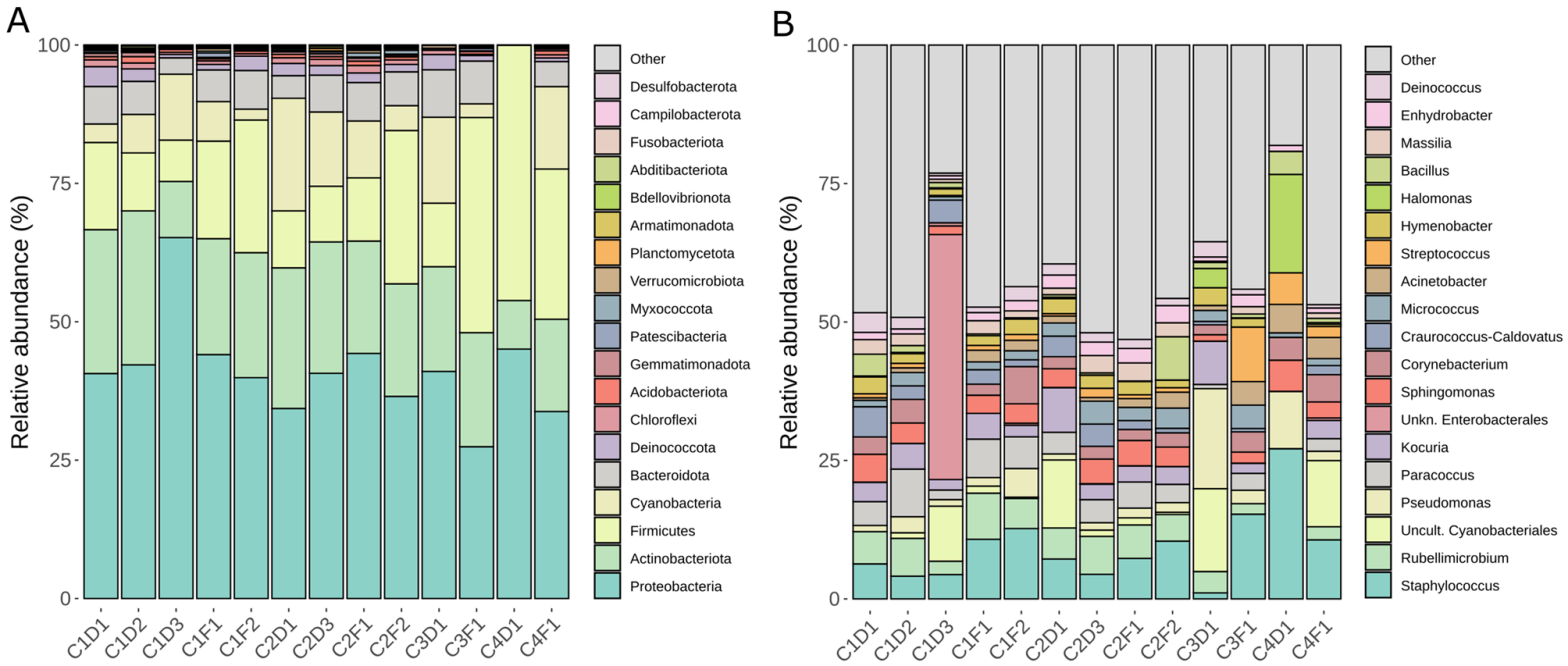
Relative abundances (%) of bacteria in the sampled cabins as deduced by high-throughput 16S rRNA gene sequencing. (**A**) Relative abundances at the phylum level. (**B**) Relative abundances at the genus level.

At the phylum level (updated according to the new nomenclature for prokaryotic phyla^32^, all the samples displayed similar bacterial profiles, with *Pseudomonadota*, *Actinomycetota* and *Bacillota* as the predominant taxa. Moreover, *Cyanobacteriota* and *Bacteroidota* were also abundant, although not in the case of sample C4D1 (inside of cabin four) (Figs. 4A and S4A), in which *Bacillota* was the predominant phylum. However, none of the phyla was statistically more abundant in any of the cabins or locations (inside/outside) according to the DESeq2 test.

At the genus level, the most similar cabins in terms of bacterial composition were cabin one and two. The genera *Staphylococcus*, *Rubellimicrobium* and *Paracoccus* were especially abundant there. Moreover, *Pseudomonas*, *Kocuria*, *Sphingomonas* and *Corynebacterium* were among the most abundant ones in the majority of the samples. Samples C1D3 (inside of cabin one), C3D1 (inside of cabin three) and C4D1 (inside of cabin four) were the most different ones (Fig. 3B). In C1D3 there was a significant higher abundance of an unknown genus within the order *Enterobacterales*. In C3D1 there was higher abundance of *Pseudomonas* and an uncultured genus within the order *Cyanobateriales*. In C4D1 *Halomonas* was especially overrepresented, whereas *Melittangium* was characteristic for samples of cabin two (Figs. 4B and S4B). Furthermore, some genera were significantly more abundant outside the cabins, such as *Oligella*, *Serratia*, *Cobetia* and *Carnobacterium*, whereas only *Providencia* was more abundant inside them according to a DESeq2 test.

**Figure 5:**
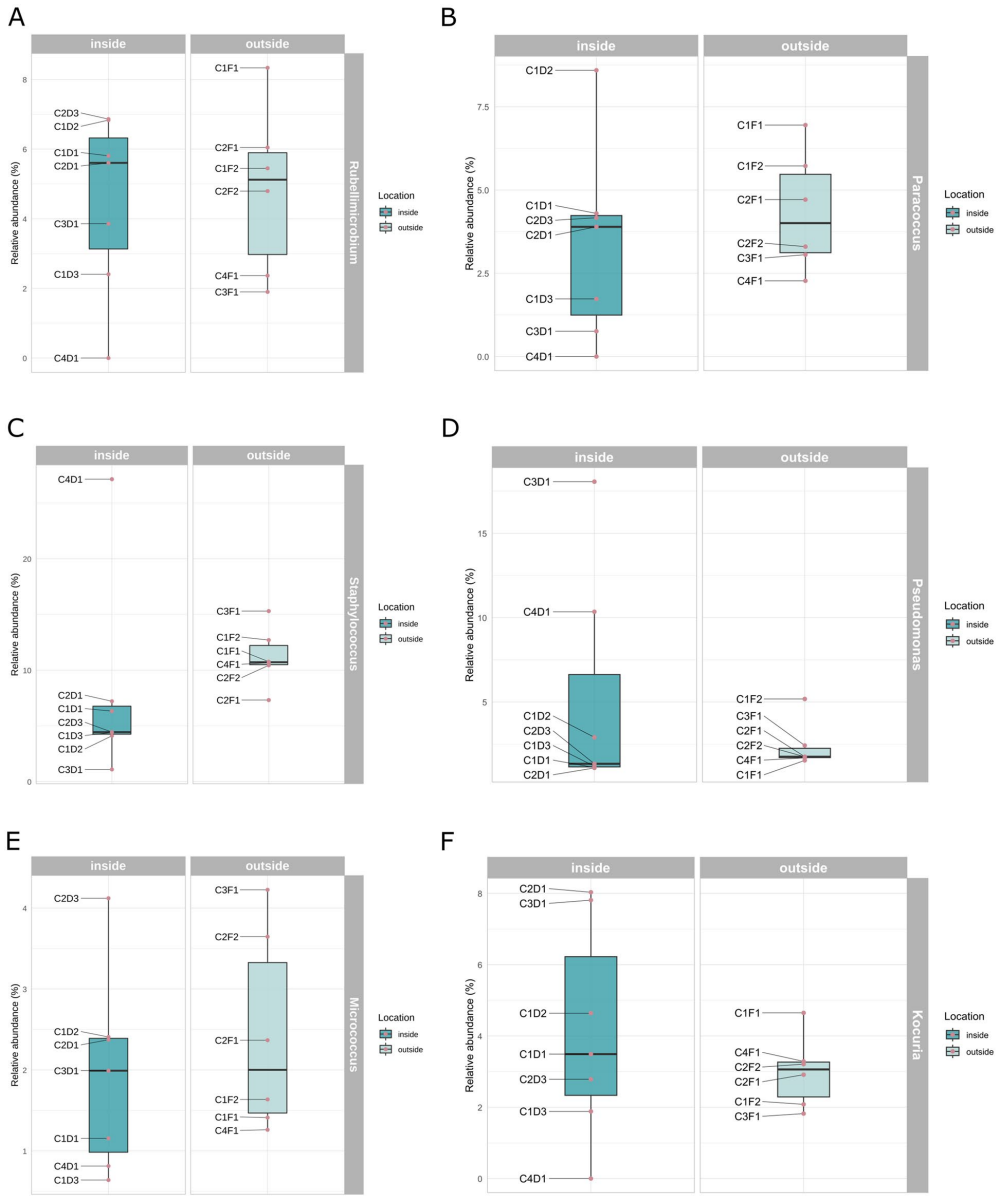
Relative abundances (%) at the genus level of specific taxa based on their abundance and relevance for the study. (**A**) Abundance of *Rubellimicrobium*. (**B**) Abundance of *Paracoccus*. (**C**) Abundance of *Staphylococcus*. (**D**) Abundance of *Pseudomonas*. (**E**) Abundance of *Micrococcus*. (**F**) Abundance of *Kocuria*.

Given the previous experience in irradiated environments, the knowledge on the natural skin microbiota, and the abundances found in the previous experiments, a selection of relevant taxa was analysed in order to determine whether there were differences in their distribution inside and outside. Moreover, the abundances of the top ten abundant genera were also plotted. That is, in alphabetical order, the genera *Corynebacterium*, *Deinococcus*, *Hymenobacter*, *Kocuria*, *Micrococcus*, *Paracoccus*, *Pseudomonas*, *Rubellimicrobium*, *Sphingomonas* and *Staphylococcus* (Figs. 5 and S5). None of the studied genera revealed any significant difference on the distribution among locations, except for *Staphylococcus* (Fig. 5C). Although *Deinococcus* was more abundant on the inner surfaces, these results were not significant according to the Wilcoxon test (Fig. S5A). This tendency was also observed in the case of *Sphingomonas* (Fig. S5D).

Finally, in order to identify the most frequent species within *Staphylococcus*, successive BLAST were performed with the most abundant ASV. Among the 61 ASV identified as *Staphylococcus*, *S. epidermitis* and *S. aureus* were between the ten more abundant species, along with *S. caprae*, *S. capitis*, *S. cohnii*, and *S. haemolyticus*.

### Biological activity assays

#### UV-radiation resistance assay

**Figure 6:**
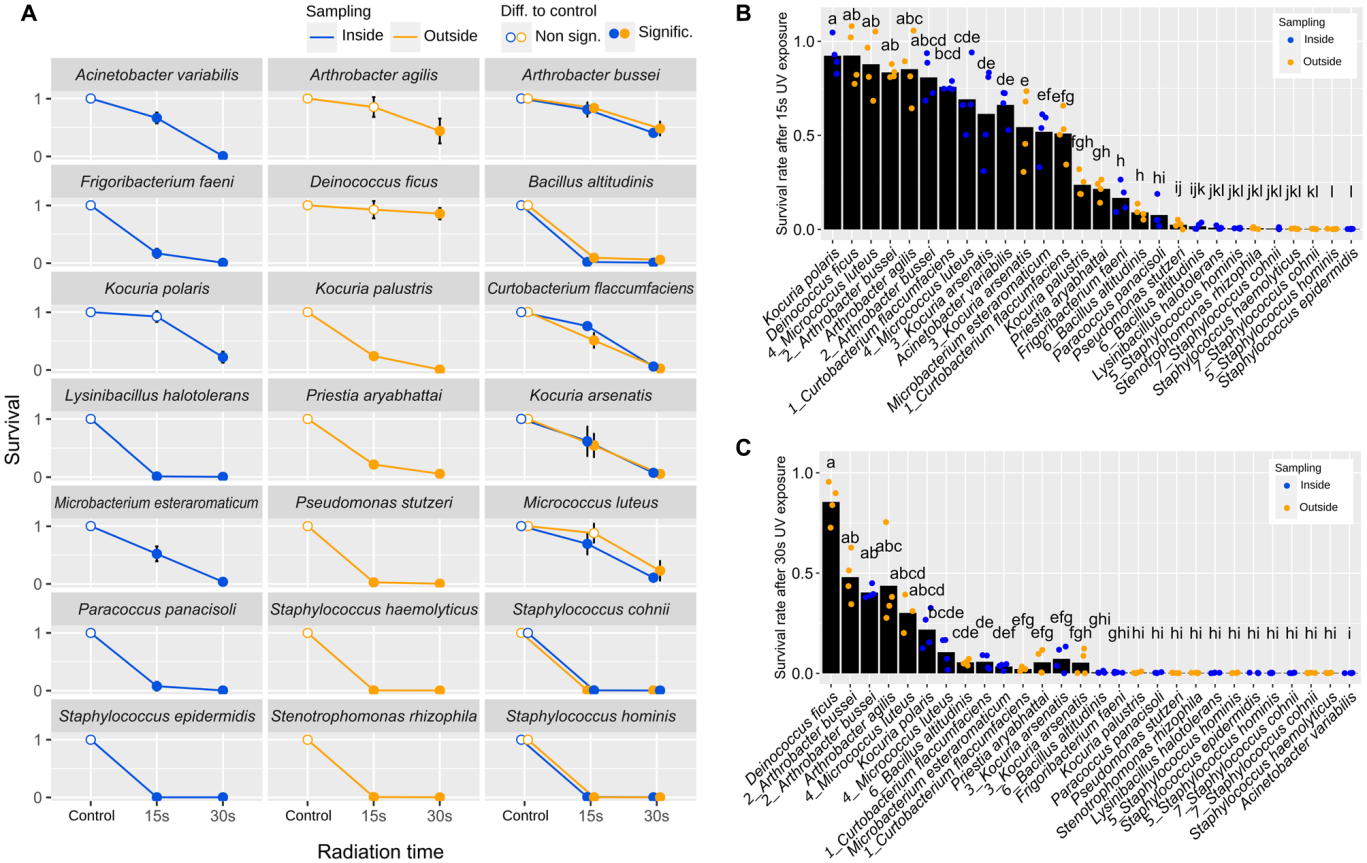
Survival rate of bacterial isolates after UV-irradiation treatment. (**A**) Lineplots showing the survival rate of cell suspensions for each of the selected isolates. Results for isolates of the same species that were taken from the inside and the outside of the UV cabins are plotted in the same facets to facilitate the comparison. The mean is depicted with a circle and standard deviation is depicted with vertical black lines. t-tests for the difference of the mean to a theoretical survival rate value of 1 were performed for 15 s and 30 s groups. Significance is stated as filled circles for *p* < 0.05. (**B**)–(**C**) Survival rate of all the strains for treatments with 15 s (**B**) or 30 s (**C**) exposure to UV irradiation. Kruskal Wallis test was performed to assess the differences among all the survival rates. Each dot represents a replicate, while the black columns represent the average. Isolates with the same letter above the column are not statistically different (*p* ≥ 0.05). Isolates taken both outside and inside samples are underlined. Results for all the panels were obtained from 4 replicates for each isolate.

The resistance to UV irradiation of the isolates from the subset of the microbial collection (Table S2) was assessed by quantifying the colony forming units (CFU) after treatments with 15 s and 30 s of exposure to UV. The survival rates were calculated by dividing the number of CFUs after irradiation between the CFUs observed in a non-exposed control replicate. Survival rates values close to 1 represented a high resistance to UV whereas close to 0 a low resistance to UV (Fig. 6A).

From the strains that were exclusively isolated inside the cabins, *Kocuria polaris* was the only one not showing a significant decrease of survival after 15 s of UV exposure. However, 30 s of exposure resulted in a sharp and significant decrease, with a survival rate close to 0. In contrast, Acinetobacter variabilis and Microbacterium esteraromaticum showed a linear decrease in the survival rate, with values after 15 s and 30 s close to 0.5 and 0 respectively, whereas, the survival rates of Frigoribacterium faeni, Lysinibacterium halotolerans, Paracoccus panacisoli and S. epidermitiss, showed a marked reduction to almost 0 after 15 s exposure to UV, revealing a high susceptibility to UV exposure.

From the strains that were exclusively isolated outside the cabins, there were two of them showing no significant decrease of survival rate after 15 s exposure to UV: *Arthrobacter agilis* and *Deinococcus ficus*. Although 30 s exposure to UV led to a significant decrease in the survival rate, these two isolates showed a difference from *K. polaris* as the values were not close to 0, suggesting a mid-resistance to the treatment. This was especially evident for *D. ficus* with a survival rate at 30 s higher than 0.75. The rest of the group formed by *Kocuria palustris*, *Priestia aryabhattai*, *Pseudomonas stutzeri*, *S. haemolyticus* and *Stenotrophomonas rhizophila* showed survival rates significantly different to the control and with values close to 0 even with 15 s of UV exposure.

Regarding the species that were isolated both inside and outside the cabins, all of them showed a similar output comparing the lineplot of the inside isolate to the outside isolate, revealing similar resistance patterns. The only exception was *Micrococcus luteus*, showing the outer strain a higher resistance to 15 s of exposure. Interestingly, the isolates of *Arthrobacter bussei* showed a similar output to the outer isolates *A agilis* and *D. ficus*, with survival rate values between 1 and 0.5 after 15 s and 30 s of UV exposure. Finally, *Curtobacterium flaccumfaciens* and *Kocuria arsenatis* also showed a considerable resistance after 15 s exposure to UV, whereas *Bacillus altitudinis*, *S. cohnii* and *S. hominis* showed a strong decrease in the resistance to UV treatment already at 15 s for both inside and outside isolates.

To further test if the UV irradiation from the cabins could shape the surface-associated microbiome leading to an enrichment of UV resistant species in the inside of the cabins, we compared in Fig. 6B,C the survival rates after 15 s and 30 s UV exposure, respectively, for all the isolates tested in Fig. 6A. However, there was no clear evidence that supported this hypothesis. Although *K. polaris* inside showed the highest survival rate after 15 s, this was not significantly different to the next four species (*D. ficus*, *M. luteus*, *A. bussei* and *A. agilis*), which were isolated outside the cabins (Fig. 6B). Moreover, a similar pattern was observed at 30 s of exposure, in which the significance group for the highest survival rates was formed by four isolates coming from the outer surfaces and two coming from the inner ones (Fig. 6C). At the strain level, *M. luteus* and *B. altitudinis* isolated from outside displayed higher survival rates than the inside isolates, whereas *C. flaccumfaciens* behaved contrarily after 15 s UV exposure. In the case of the 30 s treatment, only *B. altitudinis* behaved differently between treatments, being more resistant the outer strain.

#### Antibiotic resistance assay

To study a possible occurrence of AMR in isolates from the microbiomes of the UV cabins, we tested the resistance to antibiotics of the isolates from the subset of the microbial collection (Table S2). Quality control strains were included in all the experiments and gave the expected MIC results (in µg/ml): E. coli for amoxiclavulanicc acid (AMC; 0.5–2), doxycycline (DXT; 0.5–2) and gentamicin (GEN; 0.25–1); and S. aureus for mupirocin (MUP; 0.06–0.25), azithromycin (AZM; 0.5–2) and clindamycin (CD; 0.06–0.25). The classification of the isolates as resistant (R) or sensitive (S) to the tested antibiotics was determined according to clinical breakpoints established by EUCAST (EUCAST Clinical Breakpoint Tables v.12.0), which classifies microorganisms into susceptible at standard doses (S), susceptible increased exposure (I) or resistant (R). However, there were not available data for some species or antibiotics given the environmental origin of the tested isolates. In those cases, only the MIC values are commented and further discussed.

**Figure 7:**
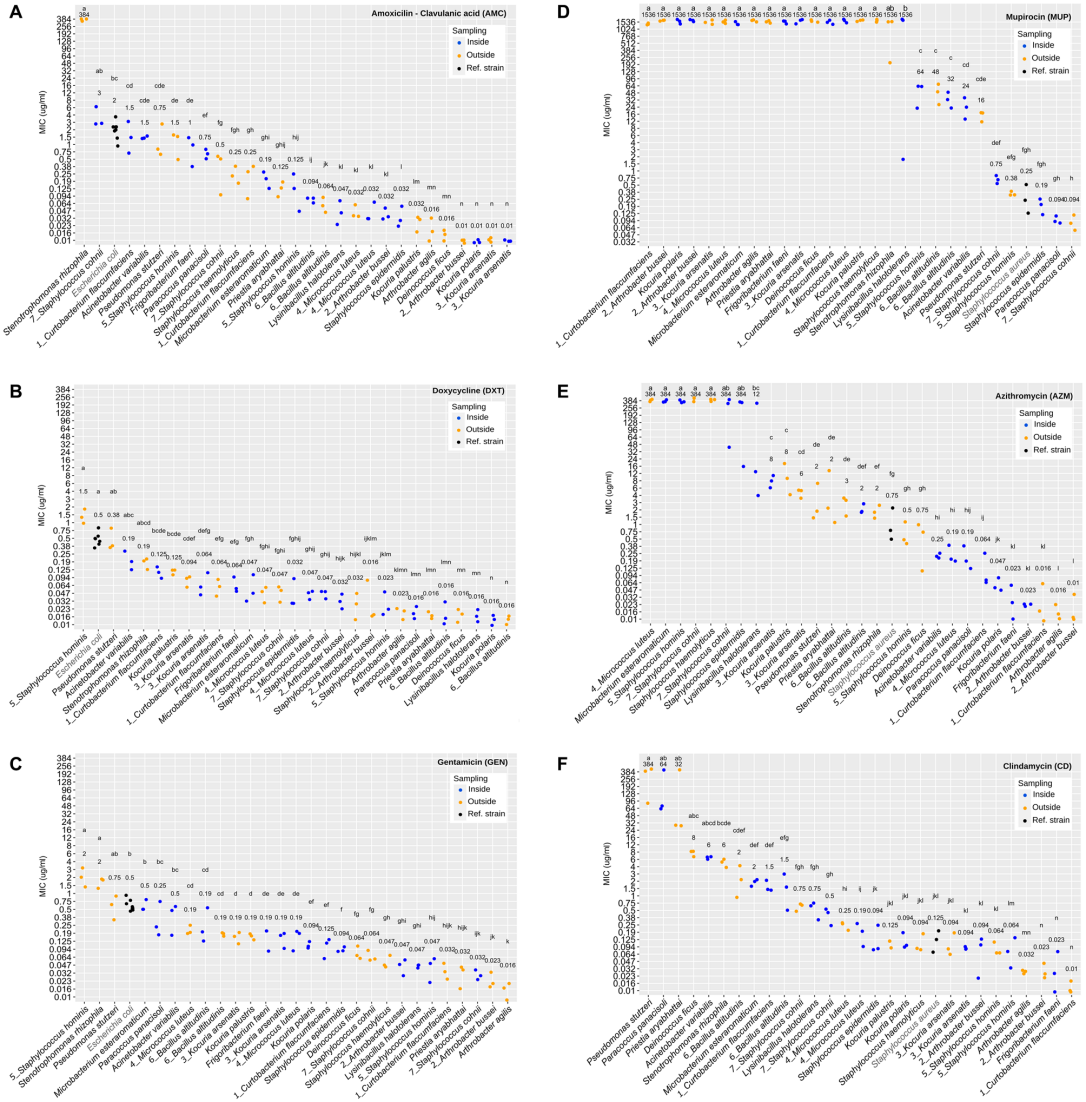
Minimum inhibitory concentration (MIC) tests displaying the resistance of bacterial isolates to antibiotics commonly used in the Dermatology Service of the HGUV. (**A**) Amoxicillin clavulanic acid (AMC). (**B**) Doxycycline (DXT). (**C**) Gentamicin (GEN). (**D**) Mupirocin (MUP). (**E**) Azithromycin (AZM). (**F**) Clindamycin (CD). Kruskal Wallis test was performed to assess for differences among MIC values. Each dot represents a replicate, while the number over the dots states the median value for each isolate. Isolates with the same letter above the column are not statistically different from each other (*p* ≥ 0.05). Isolates taken both from outside and inside the cabins are underlined. Reference strains are depicted with black dots. Expected MIC range for reference strains in µg/ml: AMC 2–8; MUP 0.06–0.25; CD 0.06–0.25; AZM 0.5–2; GEN 0.25–1; DXT 0.5–2. Results for MTSs were obtained from 3 replicates for each isolate.

The six antibiotics revealed two different patterns. On the one hand, the tested strains showed variable susceptibilities to AMC, DXT, GEN, and CD (Fig. 7A,B,C,F). In contrast, there was a clear cluster of resistant strains to MUP and AZM (Fig. 7D,E).

In the case of AMC, the majority of the tested isolates were sensitive (S or I) according to the EUCAST criteria, with the exception of *S. rhizophila* which was resistant (R, Fig. 7A). In regard to DXT, *S. hominis* appeared as I, with a MIC value between 1 and 2 µg/ml, whereas the rest of the tested strains gave MIC values below 1 (Fig. 7B). Similarly, most of the selected strains were S to GEN, with non-related species threshold stablished at 0.5 µg/ml, and 4 µg/ml for *Pseudomonas* spp. and *Acinetobacter* spp. However, in this case, *S. hominis* and *S. rhizophila* were R (Fig. 7C).

In the case of MUP and AZM, there was a significant cluster of R strains (Fig. 7D,E, respectively), with interest on *Staphylococcus* spp. in both cases: *S. haemolyticus* for MUP (R threshold at 256 µg/ml) and *S. hominis*, *S. epidermitis*, *S. cohnii* and *S. haemolyticus* for AZM (R threshold at 2 µg/ml). Moreover, both *S. cohnii* isolates displayed MIC values above the breakpoint established for *Staphylococcus* spp. for CD (Fig. 7F).

As for the strains with no registry of their resistances, their MIC values were diverse. AMC median MIC values ranged from 3 to 0.01, with the highest value at 6 µg/ml. DXT values ranged from 1.5 to 0.016, with the highest point on 4 µg/ml. GEN values varied from 2 to 0.016, being 3 µg/ml the top value. MUP values were from 1536, the top score, to 0.064 µg/ml. AZM values ranged from 384 (highest value) to 0.01 µg/ml, and CD values were from 384 (highest score) to 0.01 µg/ml.

### Phylogenetic interpretation

**Figure 8:**
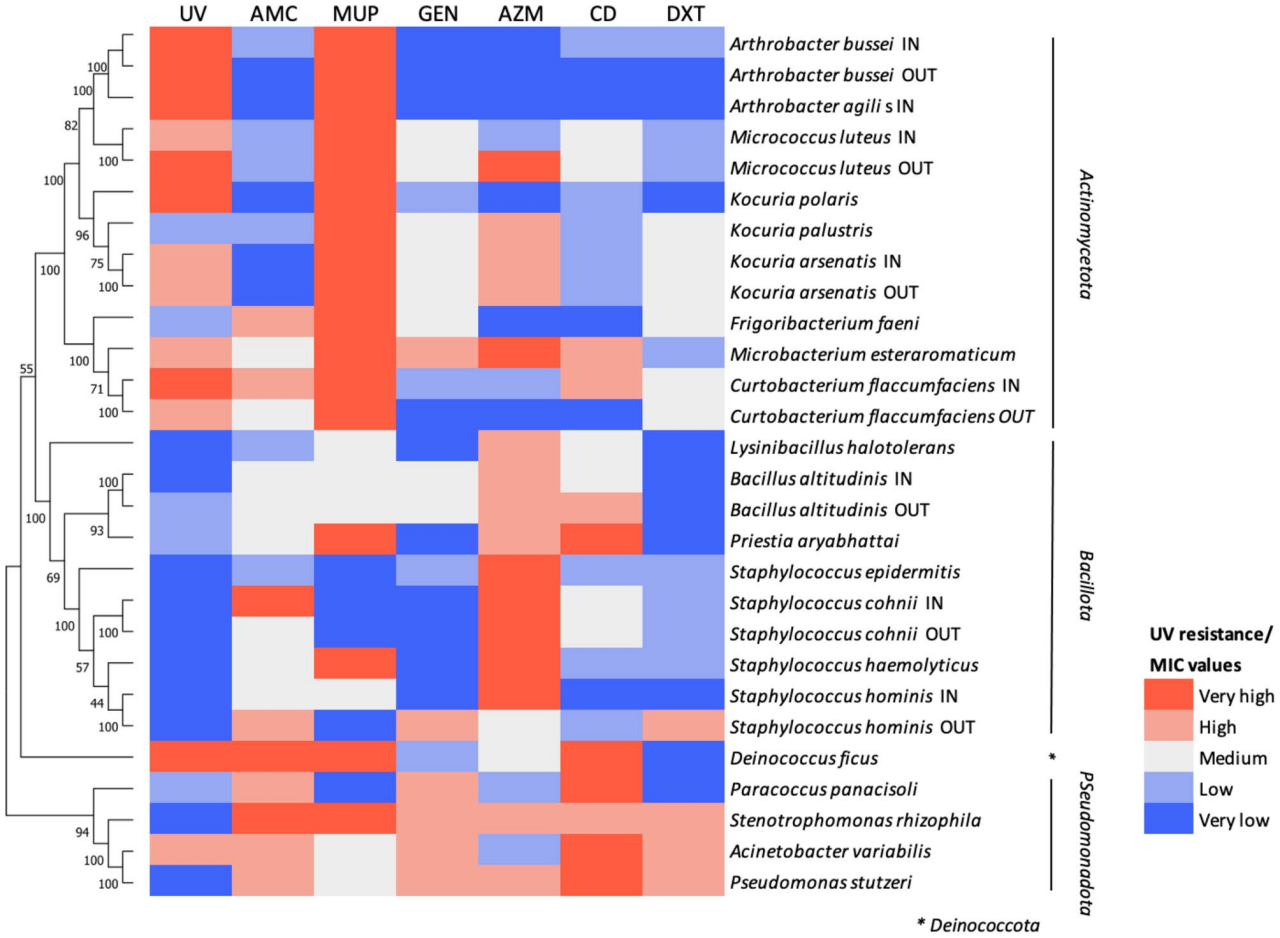
Heatmap assessing correlating the phylogenic distance of bacterial isolates and the results of biological tests (15 s of UV irradiation and antibiotic MIC tests). UV-resistance is divided in to five categories based on non-overlapping significance groups from Fig. 6. MIC values are divided into five categories based on non-overlapping significance groups from Fig. 7. Figures and significance groups [very high–high–medium–low–very low]: UV Fig. 6B [ab—e—XX—h—kl], AMC Fig. 7**A** [a—de—ghij—l—n], DXT Fig. 7**B** [ab—XX—c—XX—fh], GEN Fig. 7**C** [a—e—hi—k—mn], MUP Fig. 7**D** [ab—cde—g—i—kl], AZM Fig. 7**E** [ab—d—XX—f—hk], CD Fig. 7**F** [ab—e—XX—h—kl]. XX depicts non-used classification level.

From the phylogenetic perspective (Fig. 8), there was found out a clear tendency on *Actinomycetota* to resist UV. In contrast, species within the phylum *Bacillota* appeared as the most sensitive to this treatment. Regarding antibiotics, as stated above, there was not a significant enrichment on resistant isolates. However, MIC values were diverse. Specifically, there is interest on the resistances displayed by *staphylococci* to DXT, GEN, MUP, AZM and CD. Although the displayed values may appear as high, the available literature on clinical specimens did not catalogue most of them as resistant.

## Discussion

In the present work, we analysed the microbiomes associated to the inner and external surfaces of UV cabins used in the Dermatology Service of the Hospital General Universitario de Valencia to treat skin pathologies. First, we wanted to shed light on whether the UV irradiation shaped the microbial communities of the cabins. Second, we wanted to explore the possible correlation between UV light-resistance and resistance to antibiotics commonly used to treat skin pathologies. For this, we used a double strategy based in culture-dependent (culturomics, colony identification and biological activity tests) and independent techniques (high-throughput 16S rRNA gene sequencing).

From the culturomics point of view, the microbial profiles we found are moderately diverse and include both environmental and human associated microbial taxa (Fig. 1A). From the most abundant taxa to the least, the high abundances of *Staphylococcus*, and to a lesser extent of *Micrococcus* and *Bacillus*, are not surprising since these genera are naturally present on the human skin^33, 34^. Moreover, *Kocuria* and *Pseudomonas* have also been associated with skin disorders, such as psoriasis, and some *Arthrobacter* species are opportunistic human pathogens, such as *A. creatinolyticus* or *A. woluwensis*, or have been isolated from human clinical specimens^35–38^.

However, both *Kocuria* and *Arthrobacter* species inhabit soils, being, thus, common environmental species^2^, and the large genus *Bacillus* not only includes pathogenic species such as *B. cereus* or *B. anthracis*, but is also a typical environmental species in different natural habitats^39, 40^. Some of these genera are also known by their tolerance to biotic and abiotic stressors. Specifically, *Micrococcus* spp. and *Kocuria* spp. have been isolated from polar environments and reported to be resistant to radiation^41–44^, whereas *Arthrobacter* spp. are present in hot deserts^2, 45, 46^. Moreover, *Bacillus* spp. are well known by their tolerance to stress given their ability to form resistance spores^47^.

From the clinical point of view, we found relevant to analyze the presence of some health-threatening genera, such as *Acinetobacter* or *Pseudomonas*. Both genera have been described to have an innate adaptation ability, including the acquisition of antibiotic resistances^48–50^. Among them, *A. baumanii* and *P. aeruginosa* strains fall into the ESKAPE group of multi-drug resistant bacteria^51, 52^. Moreover, other present genera such as *Rhodococcus*, *Roseomonas* or *Stenotrophomonas* host species that cause infection to immunocompromised patients, and *Cryptococcus* spp. have been reported to cause opportunistic infections^52–56^.

Apart from the above-described ones, there is also a less abundant representation of some environmental-associated taxa, many of which have been isolated from varied environments such as *Deinococcus*, *Domibacillus*, *Pantoea* or *Sphingomonas*^57–60^. Others have been linked to isolated cases of fungaemia, bacteremia or sepsis, such as *Aureobasidium*, *Kosakonia*, *Lysinibacillus* or *Massilia*^61–64^. However, *Massilia* is also a common soil-inhabitant^65, 66^. Regarding fungi, the ones we isolated in pure culture belonged to the genera *Aureobasidium* (five isolates), *Ustilago*, *Cystobasidium*, *Rhodotorula* and *Cryptococcus* (the last four represented by just one isolate). Despite the low number of fungal isolates selected, the fact that the yeast genera *Aureobasidium*, *Rhodotorula* and *Cryptococcus* had previously been reported to inhabit different hospital facilities is in accordance with our results^67^.

The comparison of the isolation surface (inside or outside the cabins) revealed similar microbiomes. However, the existence of a cluster of exclusive genera in each location reveals some differences from the culturable point of view. The dominance of *Staphylococcus* in both isolation sources is in accordance with its widely known role in skin pathology^68^. In contrast, *Pseudomonas*, a sensitive genus, is only detected outside^69^. Curiously, all six isolates identified as *Frigoribacterium* spp. were isolated from the inner surfaces of the cabins (Fig. 1A). This genus was first described as a psychrophilic genus isolated from dust in a cattle barn in Finland^70^.

As revealed by high-throughput 16S rRNA gene sequencing, the all the cabins and locations display similar taxonomic profiles in terms of α-diversity. However, in terms β-diversity some differences are observed at the genus level (Fig. 3A,B). The fact that cabin one (working in UVA) and cabin two (working in UVB) are the most similar ones suggests that UV does not have a significant effect in shaping the microbial biocenosis in our studies (Fig. 3B).

At the phylum level, the predominant phyla found (*Pseudomonadota*, *Actinomycetota* and *Bacillota*) are in accordance with the already described profiles found in hospitals by other authors. Moreover, the comparison of the microbial profiles found in the samples at the genus level with those of hospitals is also in accordance with previous studies, particularly due to the presence of *Staphylococcus*, *Pseudomonas*, *Acinetobacter* and *Streptococcus* (Fig. 4A,B)^27, 71^. Interestingly, the presence of the genera *Rubellimicrobium*, *Deinococcus*, *Bacillus*, *Hymenobacter* and *Sphingomonas* is in line with the already described microbial communities living on solar panels^3^. This may suggest that the studied microbiomes are a combination of both highly-irradiated surfaces, such as solar panels, and hospital environments. Although, at the genus level, there are differences between both cabins and locations (Fig. 4B), the most relevant genera according to their environmental or clinical interest do not show significant differences in their distribution, with the exception of *Staphylococcus* (Fig. 5C).

Finally, the genera *Rubellimicrobium*, *Paracoccus* and *Corynebacterium* are among the most abundant genera through NGS whereas they are completely absent in the strain collection (Figs. 4B and 1A, respectively). Biases in culturing techniques are well-known and our results support the importance of combining both culture-dependent and culture-independent techniques in microbial ecology.

We tested the hypothesis that the resistance to UV irradiation of the species isolated inside the cabins would be higher than that of the species isolated outside the cabins. However, our results did not support this statement (Fig. 6). All the strains isolated exclusively from the inner surfaces showed a significant decrease in the survival rate after 15 s or 30 s of UV irradiation, with the only exception being *K. polaris* at 15 s of UV irradiation treatment. Similarly, in the group of isolates obtained exclusively from the outer surfaces there were only two strains whose survival did not decrease significantly after 15 s of UV exposure: *A. agilis* and *D. ficus*. Moreover, in the comparison of species isolated from both the inner and outer surfaces of the cabins, no relevant differences were found out. There was only one outside *M. luteus* isolate displaying higher resistance that the inner strain (Fig. 6). This suggests that, in this case, UV exposure is no causing an adaptive response for bacteria (Fig. 6A).

According to this experiment, the most resistant strains were *A. bussei, A. agilis, K. polaris, D. ficus* and *M. luteus* (Fig. 6B,C)*.* Some *Kocuria* strains have been reported as highly resistant to different types of radiation, as well as to synthesize carotenoids and encode genes related to oxidative stress^41, 44, 72–74^. Moreover, *Deinococcus* spp. have been extensively studied for its high resistance to radiation, which is a result of a combination of mechanisms such as robust DNA repair systems regulatory proteins, enzymatic and non-enzymatic antioxidant strategies^74, 75^. On its part, the genus *Arthrobacter* hosts several multi-resistant species to different abiotic stressors^76, 77^. Specifically, *A. agilis* has been reported to produce the C50 carotenoid bacterioruberin^78^. Finally, *Micrococcus* spp. have DNA repair mechanisms fundamental on their resistance to UV light^79, 80^, and *M. luteus* strains have been reported to resist high doses of gamma radiation^42^. These taxa are, thus, highly resistant to radiation and other stresses, and naturally inhabit soil and desert-like environments^2, 3^.

Even though for most of the strains the survival rate after UV treatment was significantly reduced compared to the non-irradiated control, many of them showed mid-viability after 15 s of treatment. This is the case of *A. variabilis* and *M. esteraromaticum*, isolated from the inner surfaces of the cabins, and *A. bussei, C. flaccumfaciens* and *K. arsenatis*, isolated both from the inside and the outside. Some *Acinetobacter* species have demonstrated to be able to cope with oxidative stress^81, 82^. Moreover, the genus *Microbacterium* has extensive background on UV resistance and carotenoid synthesis as well^83–85^. In contrast, less has been described about *Curtobacterium* spp., but still there are reports on their tolerance to UV^86^.

The reasons for the absence of an enrichment on resistant species inside the cabins may be varied (Fig. 6A,B). On the one hand, both surfaces are accessible to patients, which may be in contact constantly with both surfaces. This would explain also the high similarities found in terms of diversity. Moreover, the stress to which they are subjected (short pulses of UV light) may be less intense than the stress tested under laboratory conditions. From this perspective, the species tested may not represent a threaten.

As stated in the introduction, hospitals and sanitary environments increase the population of multidrug resistant pathogens. Considering that the surface of UV-cabins is constantly in contact with patients with skin pathologies, many of them with complementary treatments with antibiotics, and that the use of radiation may favor the selection of antibiotic resistant bacteria^30^, we hypothesized that the isolates taken from the inside and the outside of these cabins may present an altered susceptibility to antibiotics. In this regard, we assessed the antibiotic resistance of the isolates from Table S2 to six antibiotics widely used in dermatology: AMC, DXT, GEN, MUP, AZM and CD (Fig. 7). The classification of the strains as sensitive or resistant has been done according to the European Committee on Antimicrobial Susceptibility (EUCAST) as expressed in the instructions of the MTS.

The presence of some resistant strains is confirmed, with special interest on the genus *Staphylococcus*. In this regard, there were found isolates resistant to DXT and GEN (*S. hominis* isolated from outside), MUP (*S. haemolyticus*), AZM (both *S. cohnii* isolates, *S. haemolyticus* and *S. hominis* from inside) and CD (both *S. cohnii*). The resistance to MUP is remarkable, as this antibiotic is specifically used in the treatment of topic dermal infections by Gram-positive cocci, which also explains the resistance values displayed by most of the tested strains (which are not Gram-positive cocci). Moreover, the resistance of *S. haemolyticus* to MUP has been already reported and is mediated by the gene *mupA*^87^, and the rest of the mentioned staphylococci have reports on their multi-resistances^88–90^. There were some differences between strains isolated from different surfaces belonging to the same species, such as the case of *S. hominis*: the inside isolate is resistant to AZM whereas the outside one is resistant to DXT and GEN. As for *S. rhizophila*, our strain showed resistance to AMC, and there are reports on their multi-resistance to many antibiotics^91^. Regarding the strains for which a breakpoint has not been established, no susceptibilities can be assigned.

Taken together, the results obtained from both resistance assays (UV and antibiotics) reveal that most of microorganisms are not resistant to the antibiotics tested. Those that are, which are the staphylococci, do not show UV-resistance. The rest of the isolates we tested lack clinical interest as they are not common human pathogens. Therefore, the microbial community of the cabins is mainly composed of antibiotic-sensitive micro-organisms which display a diverse sensitivity to UV light, and a few potential pathogenic microorganisms that are sensitive to UV light (and that should thus be eliminated easily with UV-based sterilization devices). Moreover, the lack of reports of infections associated with the cabins supports a lack of substantial threat in their microbial content. However, the combination of the presence in the cabins of some microbial pathogens and the presence of antibiotic resistant genes poses an obvious potential problem linked to horizontal gene transfer (Fig. 8).

## Methods

### Sample collection

Dust samples from the inner and the outer surfaces of four UVA and UVB cabins were taken in June 2021, in the Dermatology Service of the Hospital General of Valencia, Spain. The cabins sampled were: (1) PUVA 700 Waldmann (Villingen-Schwenningen, Germany), (2) UV7001K UVA/UVB Waldmann (Villingen-Schwenningen, Germany), (3) and (4) UV7002 UVA/UVB Waldmann (Villingen-Schwenningen, Germany). Samples (in duplicate or triplicate) were obtained by scrubbing the surface with a sterile swab (FLOQSwabs™ hDNA Free, Copan Flock Technologies SRL, Brescia, Italy) and immediately stored in sterile tubes with 500 µL of Phosphate Buffer Saline (PBS) 1X until processed in the laboratory.

### Isolation of microbial strains

Samples were thoroughly shaken with vortex. As most samples were very clear, suggesting a low microbial load, 20 µL of the direct suspensions were spread on Petri dishes in duplicate, on five different culture media: TSA (composition in g/L: 15.0 tryptone, 5.0 soya peptone, 5.0 sodium chloride, 15.0 agar), Nutrient Agar (composition in g/L: peptone 5.0, meat extract 3.0, agar 15.0), Columbia Blood (catalogue number: CM0331B, ThermoFisher Scientific Inc., Massachusetts, USA), R2A (composition in g/L: 1 peptone, 0.5 yeast extract, 0.5 dextrose, 0.5 soluble starch, 0.3 dipotassium phosphate, 0.05 magnesium phosphate, 0.3 sodium pyruvate, 15.0 agar) and Yeast Mold (composition in g/L: malt extract 3.0, yeast extract 3.0, dextrose/glucose 10.0, peptone soybean 5.0, agar 15.0). Samples were incubated at 25 and 37 °C for one week. Colonies were then selected according to morphological traits, such as colour or shape, and isolated independently by re-streaking on fresh media. When pure cultures were obtained, strains were cryopreserved as glycerol stocks (12% glycerol in their isolation media) at − 80 °C until required.

### Colony identification (16S rRNA/ITS gene sequencing)

Microbial biomass from grown plates of pure cultures was suspended in 100 µL of Milli-Q sterile water. Cells were lysed by heat shock in two cycles of boiling-freezing steps. PCR was carried out for the taxonomic identification through 16S rRNA gene sequencing. Colony PCR and amplicon precipitation were carried out following the procedures previously described by Molina-Menor et al.^2^. Sequencing was performed with Sanger by Eurofins Genomics (Ebersberg, Germany). Trev tool (Staden Package, 2002) was used to manually edit 16S rRNA sequences in order to eliminate low-quality base calls. Sequences were then compared by EzBioCloud 16S rRNA BLAST tool to nucleotide databases. The sequences have been deposited under the GenBank/EMBL/DDBJ accession numbers OQ221901-OQ222055 (bacterial sequences) and OQ208835-OQ208843 (fungal sequences). Redundancy of the isolates was checked among the ones with the same identification that had been isolated from the same sample and media by Blast to Blast (https://blast.ncbi.nlm.nih.gov/Blast.cgi). The identifications are listed in Table S1.

### High-throughput 16S rRNA gene sequencing (metataxonomics)

Total DNA extraction was carried out with the DNease PowerSoil kit (MO BIO laboratories, Carlsbad, CA, USA). In order to consider potential microbial contamination of the reagents used, two negative controls consisting of pure water, instead of samples, and processed with the same kit were included. Given that very low DNA concentrations were obtained, the amplification of the 16S rRNA gene through PCR was carried out following the protocol described by Molina-Menor et al.^2^. For those samples failing to be amplified with 16S rRNA gene primers, a PCR with ITS region primers ITS3 and ITS4^92^ was carried out under the following conditions: initial step at 95 °C for 5 min, followed by 30 cycles of 30 s at 94 °C for denaturation, 30 s at 53 °C for annealing and 30 s at 72 °C for elongation, and a final step of elongation of 5 min at 72 °C. Samples with a clear amplification band in the electrophoresis gel were selected for sequencing, ensuring the representation of all four cabins and sampling points (inside and outside). The samples selected consisted of: three samples from inside and two from outside cabin one, two samples from inside and two from outside cabin two, one sample from inside and one sample from outside cabin three, and one sample from inside and one sample from outside cabin four. Both sequencing (Illumina) and the bioinformatic analysis were carried out by Darwin Bioprospecting Excellence SL (Paterna, Spain). Rarefaction curves were plotted to check the sequencing depth (Fig. S2).

### UV-radiation resistance assay

Based on the microbial identifications, a subset of isolates was selected from the collection to further perform UV irradiation and antibiotic resistance assays (from now on, sub-collection). Isolates from the most represented taxa were selected considering to have (1) a wide diversity of genera tested for each surface and (2) species that were isolated both from the inside and outside of the cabins (Table S2).

Liquid cell cultures of selected isolates were serially diluted in PBS to an OD_600_ of 10^–4^/10^–5^ in order to obtain isolated colonies after inoculation on R2A agar plates. Aliquots of 100 µl of the cultures were plated in triplicate. Two replicates were then exposed to UVC light for 15 s or 30 s, while the other replicate was the non-irradiated control. UVC irradiation was performed with a VILBER LOURMAT UV lamp emitting 354 nm light with an intensity of 340 μW/cm^2^ at 15 cm of distance. Plates were incubated at 30 °C and colonies were counted 24–72 h post inoculation depending on each strain. Number of colonies in the irradiated plates were compared to the number of colonies in the control non-irradiated plates.

### Antibiotic resistance assay

Minimum Inhibitory Concentration (MIC) tests were carried out with the commercial MIC Test Strip (MTS) by Liofilchem SRL (Roseto degli Abruzzi (TE), Italy). Amoxicillin*-clavulanic acid (2/1) (ref: 920,240, 0.016–256 mg/L), azithromycin (ref: 920,300, 0.016–256 mg/L), clindamycin (ref: 920,720, 0.016–256 mg/L), doxycycline (ref: 921,560, 0.016–256 mg/L), gentamicin (ref: 920,090, 0.016–256 mg/L) and mupirocin (ref: 920,380, 0.064–1024 mg/L) were selected among the antibiotics that are commonly used in the Dermatology Service of the Hospital General of Valencia (AMC: amoxicillin-clavulanic acid; AZM: azithromycin; CD: clindamycin; DXT: doxycycline; GEN: gentamycin; MUP: mupirocin). Growth on Mueller Hinton-agar media was tested prior to the assay in order to ensure that all the strains were able to grow on it. The experiment was carried out following the manufacturer’s instructions, using OD_600_ instead of McFarland turbidity standards to assess cell concentration. For this, PBS cell-resuspensions for each strain were diluted to OD_600_ 0.1, 0.5 and 1, and 100 μl were plated on Mueller Hinton agar plates. We selected the dilution for inoculation for each strain based on confluent cell-growth after 24, 48 and 72 h incubation at 30 °C. MIC was registered 24 h, 48 h and 72 h for each sample. The quality check strains were *Staphylococcus aureus* (ATCC 29,213–WDCM 00,131) for AZM, MUP and CD; and *Escherichia coli (*ATCC25922–WDCM 00,013) for DXT, AMC and GEN. The strains were purchased from DSMZ (German Collection of Microorganisms and Cell Cultures, Leibniz Institute, Braunschweig, Germany). The MICs for control strains showed minor changes throughout the three days that lasted each replicate, but they were always inside the expected range as referred by the manufacturer. The results were interpreted following the manufacturer’s instructions, according to the European Committee on Antimicrobial Susceptibility testing (EUCAST) (EUCAST Clinical Breakpoint Tables v.12.0).

### Phylogenetic tree

The 16S rRNA gene sequences of the type strains of the subcollection (Table S2) were retrieved from EzBioCloud (www.ezbiocloud.net). Phylogenetic analysis was carried out with MEGA11 software (v.11.0.13). Sequences were aligned with MUSCLE algorithm and the phylogenetic tree was constructed using the Neighbour Joining method^93^. The branch pattern reliability was checked with bootstrap analysis based on 100 replicates with nucleotide p-distance substitution model including transitions and transversion.

### Supplementary Information

Supplementary Information.

## Data Availability

The 16S rRNA gene sequences have been deposited under the GenBank/EMBL/DDBJ Accession Numbers OQ221901-OQ222055 (bacterial sequences) and OQ208835-OQ208843 (fungal sequences).
