# Supplementary material for: Ecology and resistance to UV light and antibiotics of microbial communities on UV cabins in the dermatology service of a Spanish hospital
Source: Sci Rep. 2023 Sep 4;13:14547. doi: 10.1038/s41598-023-40996-8 (PMC10477284; doi:10.1038/s41598-023-40996-8)
Supplement: Supplementary file 1 — Supplementary Information. [file 41598_2023_40996_MOESM1_ESM.docx]

**Ecology and resistances to UV light and antibiotics of microbial communities on UV cabins in the dermatology service of a Spanish hospital**

Esther Molina-Menor ^1^, Nicolás Carlotto ^1^, Àngela Vidal-Verdú ^1^, Amparo Pérez-Ferriols ^2^, Gemma Pérez-Pastor ^2^, and Manuel Porcar ^1,3,^*

^1^ Institute for Integrative Systems Biology (I2SysBio, University of Valencia-CSIC), Spain

^2^Servicio de Dermatología, Consorcio Hospital General de Valencia, Spain

^3^ Darwin Bioprospecting Excellence SL (Parc Científic Universitat de València, C/ CAtedràtic Agustín Escardino Benlloch 9, Paterna, Spain

*****Correspondence: manuel.porcar@uv.es

**ABSTRACT**

**Abstract:** Microorganisms colonize all possible ecological habitats, including those subjected to harsh stressors such as UV radiation. Hospitals, in particular the UV cabins used in phototherapy units, constitute an environment in which microbes are intermittently subjected to UV irradiation. This selective pressure, in addition to the frequent use of antibiotics by patients, may represent a threat in the context of the increasing problem of antimicrobial resistance. In this work, a collection of microorganisms has been established in order to study the microbiota associated to the inner and outer surfaces of UV cabins and to assess their resistance to UV light and the antibiotics frequently used in the Dermatology Service of a Spanish hospital. Our results show that UV cabins harbor a relatively diverse biocenosis dominated by typically UV-resistant microorganisms commonly found in sun-irradiated environments, such as *Kocuria*, *Micrococcus* or *Deinococcus* spp., but also clinically relevant taxa, such as *Staphylococcus* or *Pseudomonas* spp. The UV-radiation assays revealed that, although some isolates displayed some resistance, UV is not a major factor shaping the biocenosis living on the cabins, since a similar pool of resistant microorganisms was identified on the external surface of the cabins. Interestingly, some *Staphylococcus* spp. displayed resistance to one or more antibiotics, although the hospital reported no cases of antibiotic-resistance infections of the patients using the cabins. Finally, no association between UV and antibiotic resistances was found.


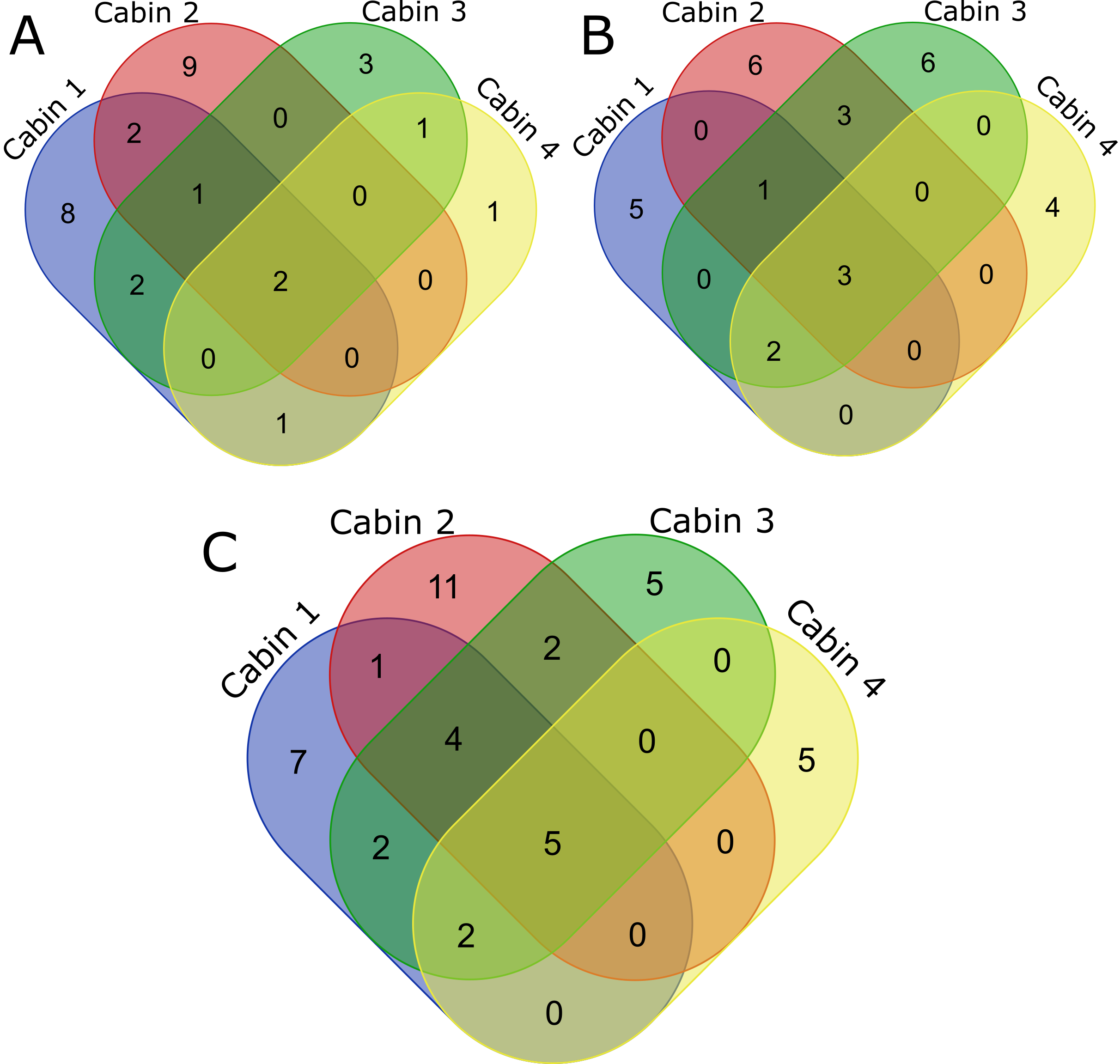


**Figure S1.** Venn diagrams showing the shared and exclusive taxa, at the genus level, in the different cabins. A) Genera isolated from inside the cabins. B) Genera isolated from outside the cabins. C) Genera regardless of the sampling site.


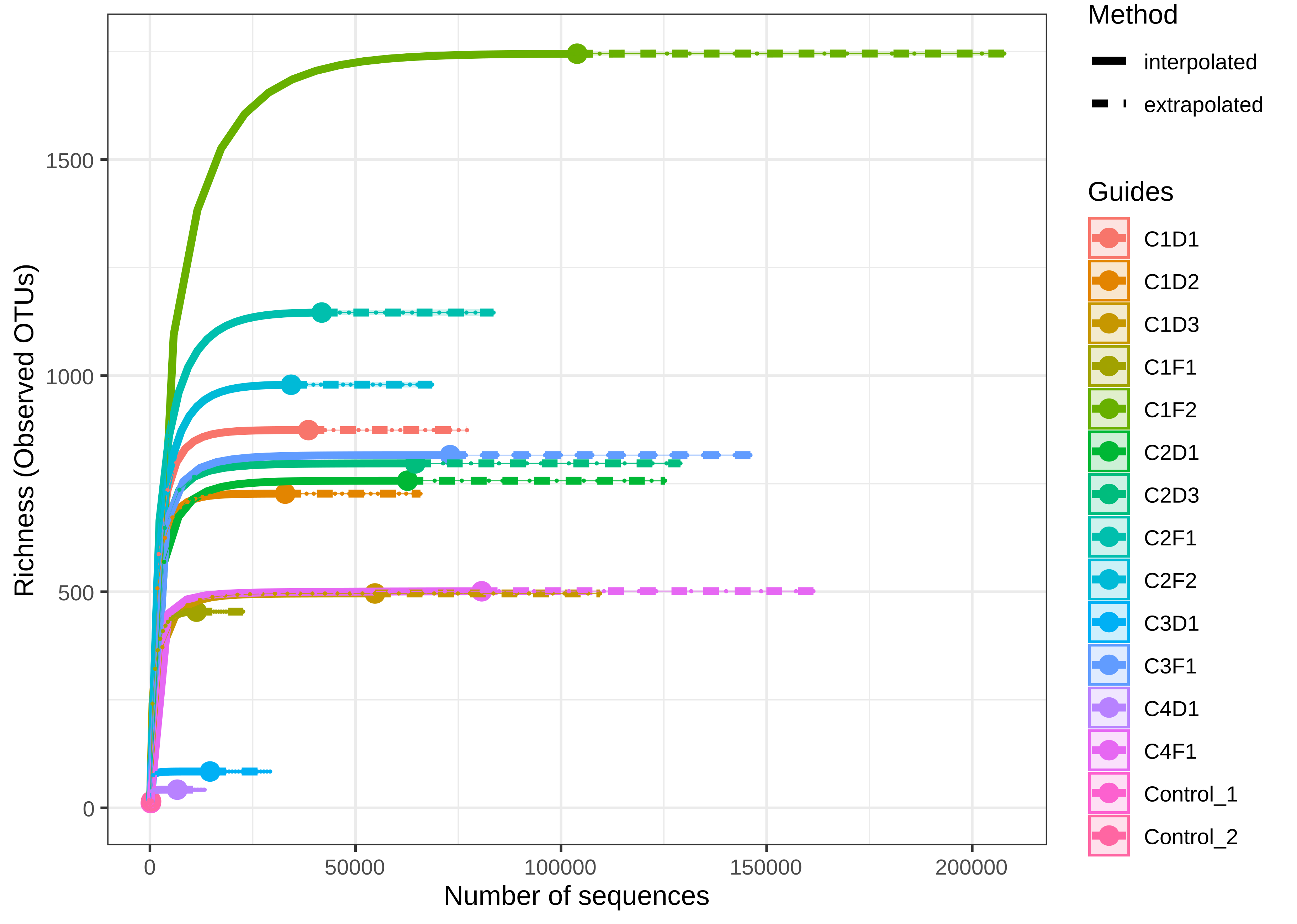


**Figure S2**. Rarefaction curve at OTU level.

A


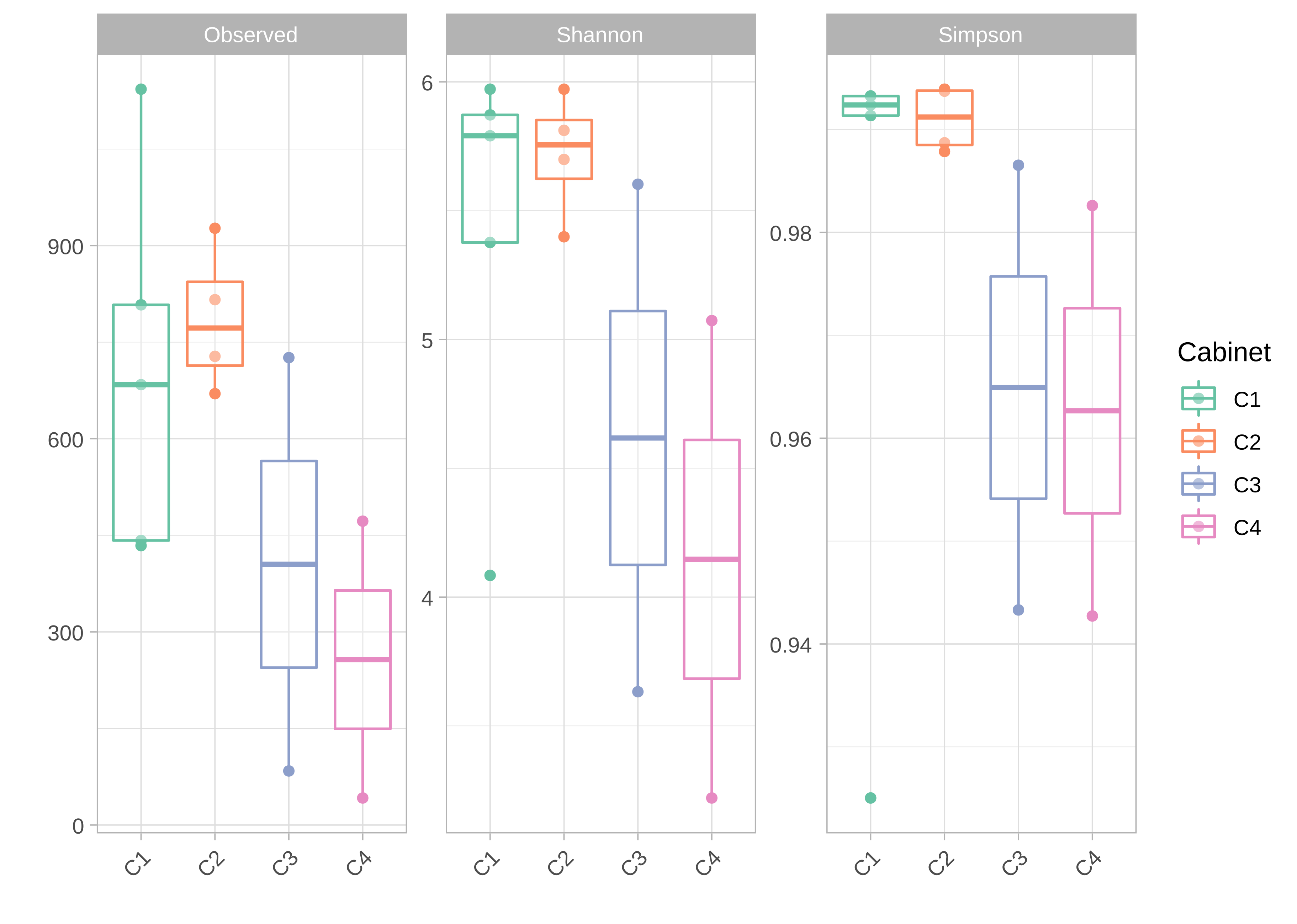


B


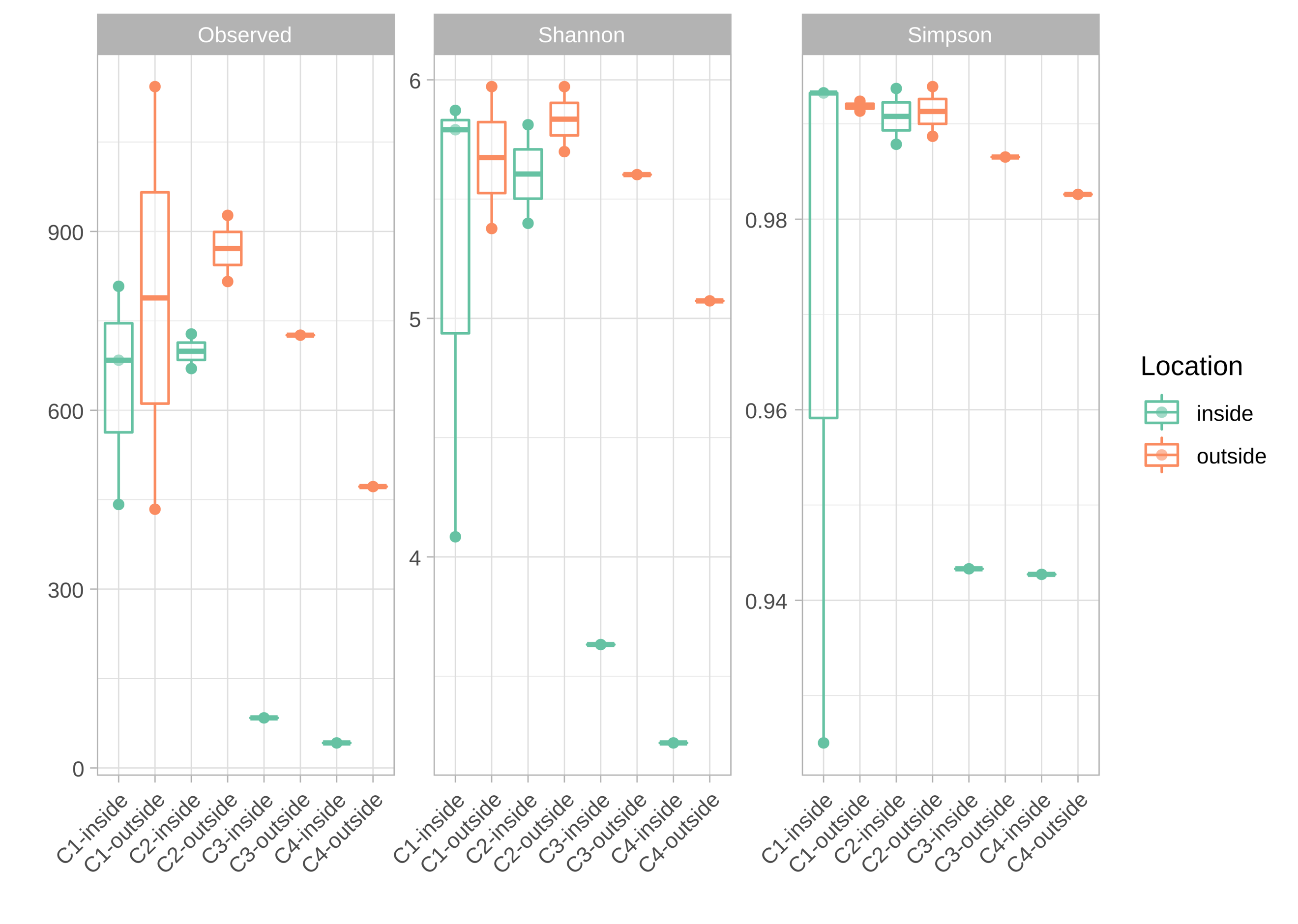


**Figure S3.** Microbial α-diversity at the ASV level (clones) observed through Wilcoxon test and measured by Shannon and Simpson indexes. A) Diversity among cabins. B) Diversity among cabins and sampling sites.

A


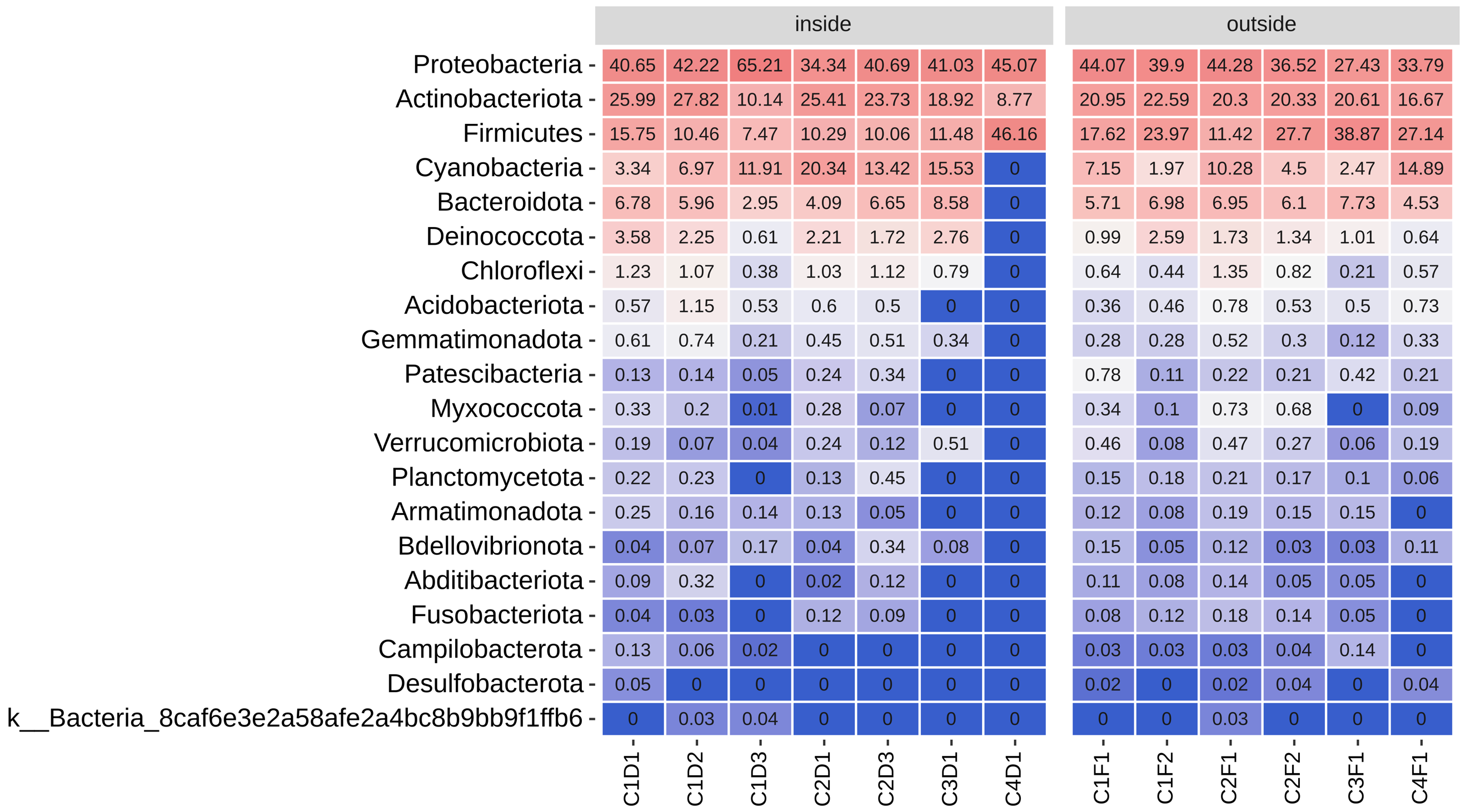


B


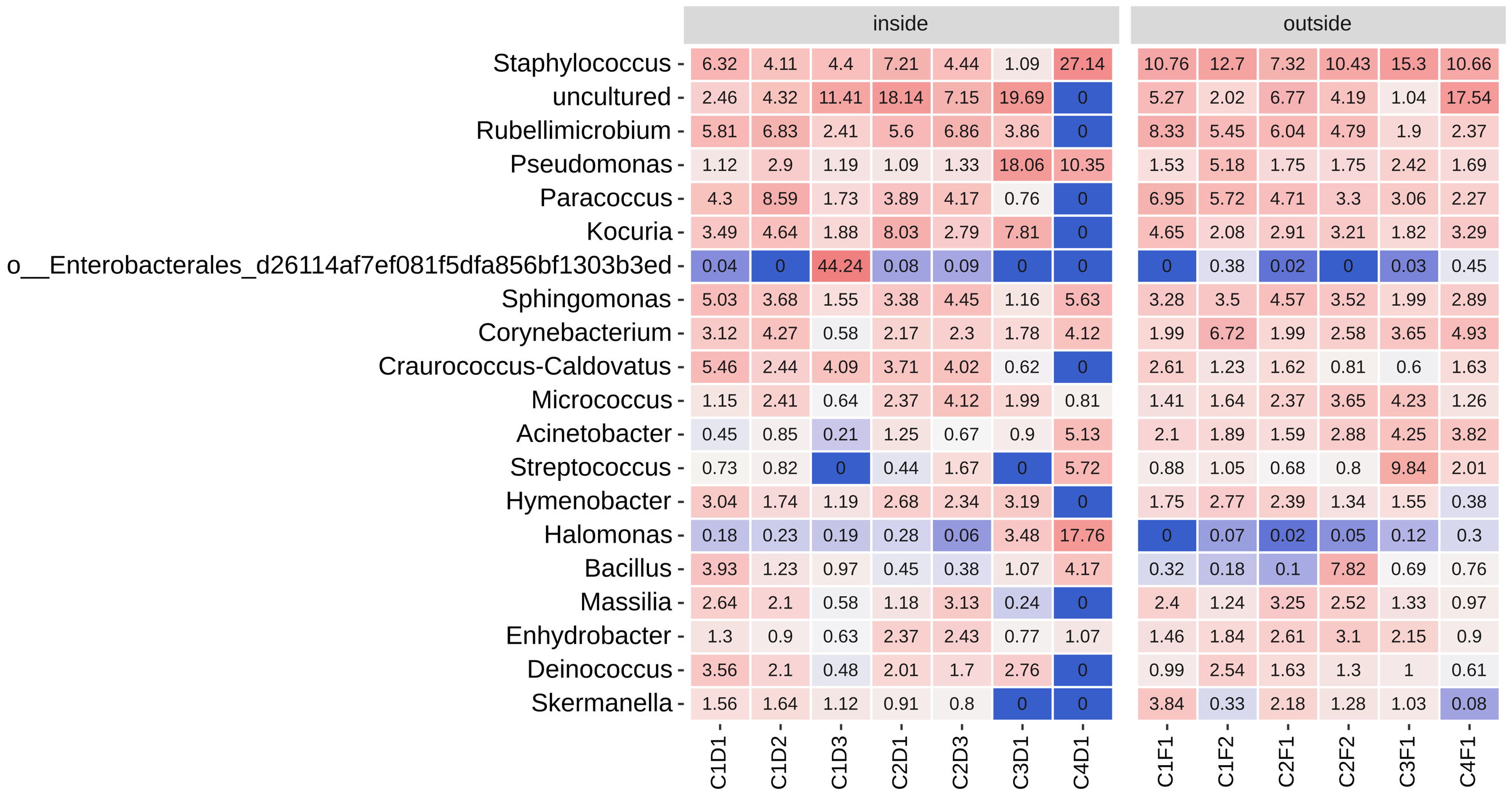


**Figure S4.** Heatmap showing the relative abundances (%) of bacteria in the sampled cabins as deduced by high-throughput 16S rRNA gene sequencing. A) Relative abundances at the phylum level. B) Relative abundances at the genus level.


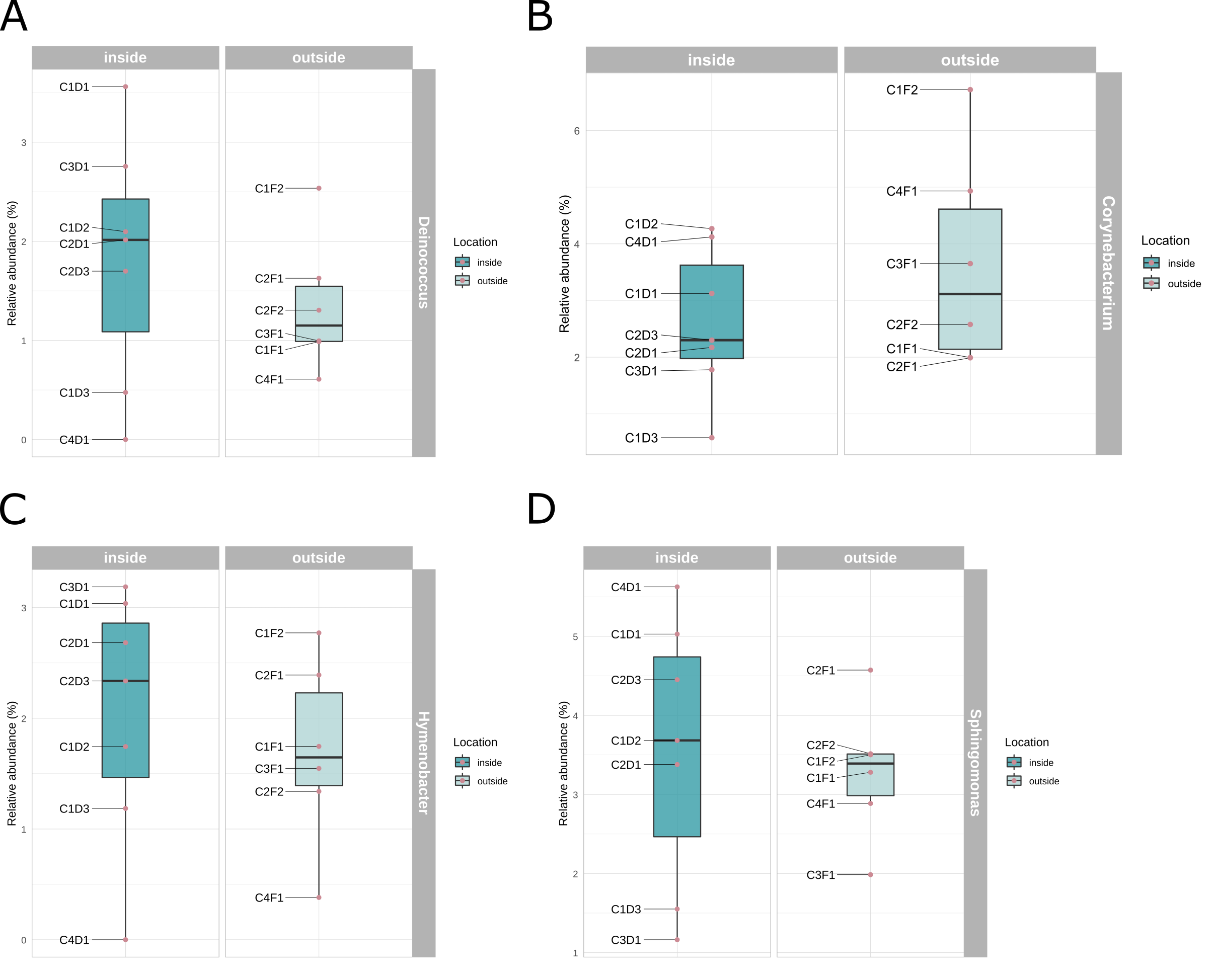


**Figure S5.** Relative abundances (%) at the genus level of specific taxa based on their abundance and relevance for the study. A) Abundance of *Deinococcus*. B) Abundance of *Corynebacterium*. C) Abundance of *Hymenobacter*. D) Abundance of *Sphingomonas*.

**Table S1.** Microbial collection identification and isolation data.

| **CODE COLLECTION** | **CABIN** | **SOURCE** | **GENUS** | **TOP HIT TYPE STRAIN** |
| --- | --- | --- | --- | --- |
| 886 | 1 | INSIDE | *Peribacillus* | *Peribacillus simplex* |
| 887 | 1 | INSIDE | *Priestia* | *Priestia megaterium* |
| 888 | 1 | OUTSIDE | *Pseudomonas* | *Pseudomonas stutzeri* |
| 889 | 1 | OUTSIDE | *Staphylococcus* | *Staphylococcus haemolyticus* |
| 890 | 1 | OUTSIDE | *Staphylococcus* | *Staphylococcus cohnii* |
| 891 | 2 | INSIDE | *Kocuria* | *Kocuria rosea* |
| 892 | 2 | INSIDE | *Staphylococcus* | *Staphylococcus borealis* |
| 893 | 2 | INSIDE | *Pseudoxanthomonas* | *Pseudoxanthomonas suwonensis* |
| 894 | 2 | OUTSIDE | *Bacillus* | *Bacillus zhangzhouensis* |
| 895 | 3 | INSIDE | *Staphylococcus* | *Staphylococcus hominis subsp. hominis* |
| 896 | 3 | OUTSIDE | *Kocuria* | *Kocuria palustris* |
| 897 | 3 | OUTSIDE | *Staphylococcus* | *Staphylococcus caprae* |
| 898 | 3 | OUTSIDE | *Micrococcus* | *Micrococcus luteus* |
| 899 | 3 | OUTSIDE | *Pseudomonas* | *Pseudomonas fulva* |
| 900 | 4 | INSIDE | *Paenibacillus* | *Paenibacillus illinoisensis* |
| 901 | 4 | INSIDE | *Staphylococcus* | *Staphylococcus cohnii* |
| 902 | 4 | OUTSIDE | *Kocuria* | *Kocuria arsenatis* |
| 903 | 1 | INSIDE | *Frigoribacterium* | *Frigoribacterium endophyticum* |
| 904 | 1 | INSIDE | *Micrococcus* | *Micrococcus luteus* |
| 905 | 1 | OUTSIDE | *Bacillus* | *Bacillus altitudinis* |
| 906 | 2 | INSIDE | *Micrococcus* | *Micrococcus luteus* |
| 907 | 2 | INSIDE | *Micrococcus* | *Micrococcus luteus* |
| 908 | 2 | OUTSIDE | *Micrococcus* | *Micrococcus luteus* |
| 909 | 2 | OUTSIDE | *Staphylococcus* | *Staphylococcus haemolyticus* |
| 910 | 2 | OUTSIDE | *Micrococcus* | *Micrococcus luteus* |
| 911 | 3 | INSIDE | *Staphylococcus* | *Staphylococcus hominis subsp. hominis* |
| 912 | 4 | INSIDE | *Staphylococcus* | *Staphylococcus hominis subsp. novobiosepticus* |
| 913 | 4 | OUTSIDE | *Micrococcus* | *Micrococcus luteus* |
| 914 | 1 | INSIDE | *Staphylococcus* | *Staphylococcus epidermidis* |
| 915 | 1 | OUTSIDE | *Priestia* | *Priestia aryabhattai* |
| 916 | 2 | INSIDE | *Staphylococcus* | *Staphylococcus hominis subsp. hominis* |
| 917 | 2 | INSIDE | *Kocuria* | *Kocuria arsenatis* |
| 918 | 2 | OUTSIDE | NID | NID |
| 919 | 2 | OUTSIDE | *Microbacterium* | *Microbacterium algeriense* |
| 920 | 3 | INSIDE | *Staphylococcus* | *Staphylococcus hominis subsp. hominis* |
| 921 | 3 | OUTSIDE | *Pseudomonas* | *Pseudomonas oryzihabitans* |
| 922 | 4 | OUTSIDE | *Roseomonas* | *Roseomonas mucosa* |
| 923 | 2 | INSIDE | *Acinetobacter* | *Acinetobacter variabilis* |
| 924 | 1 | INSIDE | *Micrococcus* | *Micrococcus luteus* |
| 925 | 1 | INSIDE | *Curtobacterium* | *Curtobacterium flaccumfaciens* |
| 926 | 1 | INSIDE | *Deinococcus* | *Deinococcus terrestris* |
| 927 | 2 | INSIDE | *Staphylococcus* | *Staphylococcus epidermidis* |
| 928 | 2 | OUTSIDE | *Metabacillus* | *Metabacillus idriensis* |
| 929 | 4 | OUTSIDE | *Staphylococcus* | *Staphylococcus hominis subsp. novobiosepticus* |
| 930 | 2 | OUTSIDE | *Pseudomonas* | *Pseudomonas kuykendallii* |
| 931 | 3 | INSIDE | *Bacillus* | *Bacillus licheniformis* |
| 932 | 3 | INSIDE | *Lysinibacillus* | *Lysinibacillus halotolerans* |
| 933 | 3 | INSIDE | *Micrococcus* | *Micrococcus luteus* |
| 934 | 3 | OUTSIDE | *Deinococcus* | *Deinococcus ficus* |
| 935 | 3 | INSIDE | *Kocuria* | *Kocuria arsenatis* |
| 936 | 3 | OUTSIDE | *Paracoccus* | *Paracoccus chinensis* |
| 937 | 3 | OUTSIDE | *Pantoea* | *Pantoea conspicua* |
| 938 | 3 | OUTSIDE | *Bacillus* | *Bacillus manliponensis* |
| 939 | 3 | OUTSIDE | *Paracoccus* | *Paracoccus niistensis* |
| 940 | 4 | INSIDE | *Kocuria* | *Kocuria turfanensis* |
| 941 | 4 | OUTSIDE | *Kocuria* | *Kocuria arsenatis* |
| 942 | 1 | INSIDE | *Metabacillus* | *Metabacillus idriensis* |
| 943 | 1 | OUTSIDE | *Bacillus* | *Bacillus aerius* |
| 944 | 1 | OUTSIDE | *Pseudomonas* | *Pseudomonas oryzihabitans* |
| 945 | 2 | INSIDE | *Planococcus* | *Planococcus glaciei* |
| 946 | 2 | INSIDE | *Kocuria* | *Kocuria rosea* |
| 947 | 2 | OUTSIDE | *Micrococcus* | *Micrococcus luteus* |
| 948 | 2 | OUTSIDE | *Staphylococcus* | *Staphylococcus edaphicus* |
| 949 | 3 | INSIDE | *Robertmurraya* | *Robertmurraya siralis* |
| 950 | 3 | INSIDE | *Bacillus* | *Bacillus altitudinis* |
| 951 | 3 | OUTSIDE | *Micrococcus* | *Micrococcus luteus* |
| 952 | 3 | OUTSIDE | *Metabacillus* | *Metabacillus halosaccharovorans* |
| 953 | 3 | OUTSIDE | *Staphylococcus* | *Staphylococcus equorum subsp. equorum* |
| 954 | 4 | INSIDE | *Bacillus* | *Bacillus pseudomycoides* |
| 955 | 4 | INSIDE | *Staphylococcus* | *Staphylococcus saprophyticus subsp. saprophyticus* |
| 956 | 4 | OUTSIDE | *Staphylococcus* | *Staphylococcus edaphicus* |
| 957 | 4 | OUTSIDE | *Cytobacillus* | *Cytobacillus firmus* |
| 958 | 1 | INSIDE | *Kocuria* | *Kocuria rosea* |
| 959 | 1 | INSIDE | *Micrococcus* | *Micrococcus luteus* |
| 960 | 1 | INSIDE | *Arthrobacter* | *Arthrobacter bussei* |
| 961 | 1 | INSIDE | *Micrococcus* | *Micrococcus luteus* |
| 962 | 2 | INSIDE | *Arthrobacter* | *Arthrobacter bussei* |
| 963 | 1 | OUTSIDE | *Aureobasidium* | *Aureobasidium namibiae* |
| 964 | 2 | INSIDE | *Acinetobacter* | *Acinetobacter variabilis* |
| 965 | 2 | OUTSIDE | *Psychrobacillus* | *Psychrobacillus psychrodurans* |
| 966 | 3 | INSIDE | *Micrococcus* | *Micrococcus luteus* |
| 967 | 3 | OUTSIDE | *Pseudomonas* | *Pseudomonas oryzihabitans* |
| 968 | 3 | OUTSIDE | NID | NID |
| 969 | 3 | OUTSIDE | *Planococcus* | *Planococcus okeanokoites* |
| 970 | 4 | OUTSIDE | *Kocuria* | *Kocuria arsenatis* |
| 971 | 4 | OUTSIDE | *Staphylococcus* | *Staphylococcus borealis* |
| 972 | 4 | OUTSIDE | *Ustilago* | *Ustilago shanxiensis* |
| 973 | 4 | INSIDE | *Staphylococcus* | *Staphylococcus hominis subsp. novobiosepticus* |
| 974 | 4 | OUTSIDE | *Micrococcus* | *Micrococcus luteus* |
| 975 | 1 | INSIDE | *Domibacillus* | *Domibacillus indicus* |
| 976 | 1 | INSIDE | *Streptomyces* | *Streptomyces flavovirens* |
| 977 | 1 | INSIDE | *Frigoribacterium* | *Frigoribacterium faeni* |
| 978 | 1 | OUTSIDE | *Streptomyces* | *Streptomyces ambofaciens* |
| 979 | 1 | OUTSIDE | *Priestia* | *Priestia aryabhattai* |
| 980 | 1 | OUTSIDE | *Bacillus* | *Bacillus altitudinis* |
| 981 | 2 | INSIDE | *Sphingomonas* | *Sphingomonas aerolata* |
| 982 | 2 | INSIDE | *Massilia* | *Massilia dura* |
| 983 | 2 | OUTSIDE | *Pseudomonas* | *Pseudomonas oryzihabitans* |
| 984 | 2 | OUTSIDE | *Staphylococcus* | *Staphylococcus argenteus* |
| 985 | 2 | OUTSIDE | *Arthrobacter* | *Arthrobacter agilis* |
| 986 | 2 | OUTSIDE | *Neobacillus* | *Neobacillus niacini* |
| 987 | 3 | OUTSIDE | NID | NID |
| 988 | 4 | INSIDE | *Staphylococcus* | *Staphylococcus cohnii* |
| 989 | 4 | OUTSIDE | *Kocuria* | *Kocuria arsenatis* |
| 990 | 4 | OUTSIDE | *Pseudomonas* | *Pseudomonas kuykendallii* |
| 991 | 1 | INSIDE | *Cryptococcus* | *[Cryptococcus] albidus var. kuetzingii* |
| 992 | 2 | INSIDE | *Cystobasidium* | *Cystobasidium slooffiae* |
| 993 | 2 | INSIDE | *Massilia* | *Massilia arvi* |
| 994 | 2 | INSIDE | *Rhodotorula* | *Rhodotorula mucilaginosa* |
| 995 | 2 | INSIDE | *Saccharibacillus* | *Saccharibacillus qingshengii* |
| 996 | 2 | OUTSIDE | *Erwinia* | *Erwinia endophytica* |
| 997 | 3 | INSIDE | *Staphylococcus* | *Staphylococcus hominis subsp. hominis* |
| 998 | 4 | INSIDE | *Paenibacillus* | *Paenibacillus illinoisensis* |
| 999 | 3 | OUTSIDE | *Aureobasidium* | *Aureobasidium namibiae* |
| 1000 | 4 | OUTSIDE | *Kocuria* | *Kocuria arsenatis* |
| 1001 | 4 | OUTSIDE | *Novosphingobium* | *Novosphingobium silvae* |
| 1002 | 1 | INSIDE | *Aureobasidium* | *Aureobasidium namibiae* |
| 1003 | 1 | OUTSIDE | *Moraxella* | *Moraxella osloensis* |
| 1004 | 2 | INSIDE | *Massilia* | *Massilia arvi* |
| 1005 | 2 | INSIDE | *Saccharibacillus* | *Saccharibacillus qingshengii* |
| 1006 | 2 | INSIDE | *Acinetobacter* | *Acinetobacter variabilis* |
| 1007 | 2 | OUTSIDE | *Massilia* | *Massilia agri* |
| 1008 | 2 | OUTSIDE | *Stenotrophomonas* | *Stenotrophomonas rhizophila* |
| 1009 | 3 | OUTSIDE | *Kosakonia* | *Kosakonia cowanii* |
| 1010 | 3 | OUTSIDE | *Staphylococcus* | *Staphylococcus hominis subsp. novobiosepticus* |
| 1011 | 3 | OUTSIDE | *Arthrobacter* | *Arthrobacter agilis* |
| 1012 | 4 | INSIDE | *Curtobacterium* | *Curtobacterium citreum* |
| 1013 | 4 | INSIDE | *Curtobacterium* | *Curtobacterium oceanosedimentum* |
| 1014 | 4 | OUTSIDE | *Kocuria* | *Kocuria arsenatis* |
| 1015 | 4 | OUTSIDE | *Novosphingobium* | *Novosphingobium silvae* |
| 1016 | 1 | INSIDE | *Microbacterium* | *Microbacterium esteraromaticum* |
| 1017 | 1 | INSIDE | *Arthrobacter* | *Arthrobacter bussei* |
| 1018 | 1 | INSIDE | *Frigoribacterium* | *Frigoribacterium faeni* |
| 1019 | 1 | OUTSIDE | *Micrococcus* | *Micrococcus luteus* |
| 1020 | 1 | OUTSIDE | *Kocuria* | *Kocuria arsenatis* |
| 1021 | 1 | OUTSIDE | *Cellulomonas* | *Cellulomonas pakistanensis* |
| 1022 | 1 | OUTSIDE | *Bacillus* | *Bacillus toyonensis* |
| 1023 | 1 | OUTSIDE | NID | NID |
| 1024 | 2 | INSIDE | *Staphylococcus* | *Staphylococcus epidermidis* |
| 1025 | 2 | INSIDE | *Paracoccus* | *Paracoccus halotolerans* |
| 1026 | 2 | INSIDE | *Staphylococcus* | *Staphylococcus cohnii* |
| 1027 | 2 | INSIDE | *Arthrobacter* | *Arthrobacter bussei* |
| 1028 | 2 | INSIDE | *Kocuria* | *Kocuria polaris* |
| 1029 | 2 | OUTSIDE | *Fredinandcohnia* | *Fredinandcohnia onubensis* |
| 1030 | 2 | OUTSIDE | NID | NID |
| 1031 | 2 | OUTSIDE | *Pseudomonas* | *Pseudomonas stutzeri* |
| 1032 | 3 | INSIDE | *Microbacterium* | *Microbacterium esteraromaticum* |
| 1033 | 3 | INSIDE | *Frigoribacterium* | *Frigoribacterium faeni* |
| 1034 | 3 | INSIDE | *Rhodococcus* | *Rhodococcus corynebacterioides* |
| 1035 | 3 | OUTSIDE | *Pseudomonas* | *Pseudomonas oryzihabitans* |
| 1036 | 3 | OUTSIDE | *Aureobasidium* | *Aureobasidium namibiae* |
| 1037 | 4 | INSIDE | *Staphylococcus* | *Staphylococcus cohnii* |
| 1038 | 4 | OUTSIDE | *Kocuria* | *Kocuria palustris* |
| 1039 | 4 | OUTSIDE | *Aureobasidium* | *Aureobasidium namibiae* |
| 1040 | 3 | OUTSIDE | *Arthrobacter* | *Arthrobacter bussei* |
| 1041 | 3 | INSIDE | *Micrococcus* | *Micrococcus luteus* |
| 1042 | 4 | OUTSIDE | *Cytobacillus* | *Cytobacillus horneckiae* |
| 1043 | 4 | OUTSIDE | *Kocuria* | *Kocuria arsenatis* |
| 1044 | 2 | INSIDE | *Cellulomonas* | *Cellulomonas cellasea* |
| 1045 | 1 | INSIDE | *Paracoccus* | *Paracoccus panacisoli* |
| 1046 | 1 | INSIDE | *Priestia* | *Priestia endophytica* |
| 1047 | 2 | INSIDE | *Staphylococcus* | *Staphylococcus hominis subsp. hominis* |
| 1048 | 1 | OUTSIDE | *Mixta* | *Mixta calida* |
| 1049 | 3 | INSIDE | *Frigoribacterium* | *Frigoribacterium faeni* |
| 1050 | 3 | INSIDE | *Frigoribacterium* | *Frigoribacterium faeni* |
| 1051 | 2 | OUTSIDE | *Staphylococcus* | *Staphylococcus edaphicus* |
| 1052 | 3 | OUTSIDE | *Curtobacterium* | *Curtobacterium flaccumfaciens* |
| 1053 | 1 | OUTSIDE | *Pseudomonas* | *Pseudomonas stutzeri* |
| 1054 | 3 | OUTSIDE | *Neobacillus* | *Neobacillus niacini* |

**Table S2.** Selected strains for biological activity assays.

| **CODE COLLECTION** | **ID** | **SOURCE** |
| --- | --- | --- |
| 889 | *Staphylococcus haemolyticus* | Outside |
| 890 | *Staphylococcus cohnii* | Outside |
| 896 | *Kocuria palustris* | Outside |
| 901 | *Staphylococcus cohnii* | Inside |
| 906 | *Micrococcus luteus* | Inside |
| 912 | *Staphylococcus hominis* | Inside |
| 925 | *Curtobacterium flaccumfaciens* | Inside |
| 927 | *Staphylococcus epidermidis* | Inside |
| 929 | *Staphylococcus hominis* | Outside |
| 932 | *Lysinibacillus halotolerans* | Inside |
| 934 | *Deinococcus ficus* | Outside |
| 935 | *Kocuria arsenatis* | Inside |
| 950 | *Bacillus altitudinis* | Inside |
| 964 | *Acinetobacter variabilis* | Inside |
| 977 | *Frigoribacterium faeni* | Inside |
| 979 | *Priestia aryabhattai* | Outside |
| 980 | *Bacillus altitudinis* | Outside |
| 1008 | *Stenotrophomonas rhizophila* | Outside |
| 1011 | *Arthrobacter agilis* | Outside |
| 1016 | *Microbacterium esteraromaticum* | Inside |
| 1019 | *Micrococcus luteus* | Outside |
| 1020 | *Kocuria arsenatis* | Outside |
| 1027 | *Arthrobacter bussei* | Inside |
| 1028 | *Kocuria polaris* | Inside |
| 1040 | *Arthrobacter bussei* | Outside |
| 1045 | *Paracoccus panacisoli* | Inside |
| 1052 | *Curtobacterium flaccumfaciens* | Outside |
| 1053 | *Pseudomonas stutzeri* | Outside |
